# Interleukin-1 contributes to clonal expansion and progression of bone marrow fibrosis in JAK2V617F-induced myeloproliferative neoplasm

**DOI:** 10.1038/s41467-022-32928-3

**Published:** 2022-09-13

**Authors:** Mohammed Ferdous-Ur Rahman, Yue Yang, Bao T. Le, Avik Dutta, Julia Posyniak, Patrick Faughnan, Mohammad A. Sayem, Nadine S. Aguilera, Golam Mohi

**Affiliations:** 1 Department of Biochemistry and Molecular Genetics, University of Virginia School of Medicine, Charlottesville, VA 22908 USA; 2 Department of Pathology, University of Virginia School of Medicine, Charlottesville, VA 22908 USA; 3 University of Virginia Cancer Center, Charlottesville, VA 22908 USA

**Keywords:** Myeloproliferative disease, Myeloproliferative disease

## Abstract

Chronic inflammation is frequently associated with myeloproliferative neoplasms (MPN), but the role of inflammation in the pathogenesis of MPN remains unclear. Expression of the proinflammatory cytokine interleukin-1 (IL-1) is elevated in patients with MPN as well as in Jak2V617F knock-in mice. Here, we show that genetic deletion of IL-1 receptor 1 (IL-1R1) normalizes peripheral blood counts, reduces splenomegaly and ameliorates bone marrow fibrosis in homozygous Jak2V617F mouse model of myelofibrosis. Deletion of IL-1R1 also significantly reduces Jak2V617F mutant hematopoietic stem/progenitor cells. Exogenous administration of IL-1β enhances myeloid cell expansion and accelerates the development of bone marrow fibrosis in heterozygous Jak2V617F mice. Furthermore, treatment with anti-IL-1R1 antibodies significantly reduces leukocytosis and splenomegaly, and ameliorates bone marrow fibrosis in homozygous Jak2V617F mice. Collectively, these results suggest that IL-1 signaling plays a pathogenic role in MPN disease progression, and targeting of IL-1R1 could be a useful strategy for the treatment of myelofibrosis.

## Abstract (web summary)

Chronic inflammation is frequently observed in patients with myeloproliferative neoplasms (MPN). Here the authors observe elevated levels of IL-1α and IL-1β in MPN patients and show that IL-1 contributes to clonal expansion and bone marrow fibrosis in a mouse model of MPN, proposing targeting of IL1R1 as therapeutic intervention.

## Introduction

Myeloproliferative neoplasms (MPN) including polycythemia vera (PV), essential thrombocythemia (ET), and myelofibrosis (MF) are a group of clonal hematopoietic stem cell derived myeloid malignancies characterized by aberrant production of myeloid, erythroid or megakaryocytic lineage cells. JAK2V617F is the most common somatic driver mutation associated with MPN^1^. Interestingly, JAK2V617F mutation can also be detected in healthy individuals with clonal hematopoiesis of indeterminate potential (CHIP) who do not exhibit overt changes in blood leukocytes, erythrocytes and platelets^2^. It has been suggested that the JAK2V617F mutation occurs in a single hematopoietic stem cell (HSC) decades before MPN diagnosis^3^. The JAK2V617F mutation can also be detected in the utero, suggesting the existence of this somatic driver mutation early in life^4^. The JAK inhibitor ruxolitinib can alleviate constitutional symptoms but it does not eliminate malignant clones or offer significant reduction in bone marrow fibrosis in patients with MPN/MF^5^. This suggests that other factors might be involved in association with JAK2 mutation in clonal expansion and initiation/progression of MPN disease.

Chronic inflammation is frequently observed in MPN^6^. Patients with MPN often exhibit increased levels of inflammatory cytokines^6,7^. Gene expression analysis also confirms enrichment of inflammatory and immune system gene signatures in MPN hematopoietic cells^8^. Prior history of autoimmune diseases increases the risk of MPN development^9^. Furthermore, inflammation has been associated with progression to bone marrow fibrosis^7^. However, the key inflammatory signaling involved in the initiation and progression of MPN remains poorly understood.

Interleukin 1 (IL-1) is a major regulator of inflammation^10,11^. IL-1 consists of two related cytokines IL-1α and IL-1β with overlapping functions^10,11^. IL-1α and IL-1β bind to the IL-1 receptor 1 (IL-1R1) to initiate downstream signaling^10,11^. While IL-1 or IL-1R1 is dispensable for steady state hematopoiesis^12,13^, acute or chronic exposure of IL-1 drives myeloid differentiation of hematopoietic stem cells (HSC)^14^. Elevated levels of IL-1 have been observed in patients with MPN and are strongly correlated with myelofibrosis^6,7,15,16^. While IL-1 has been implicated in various inflammatory pathophysiological conditions including cardiovascular diseases, lung fibrosis, cancer and autoimmune diseases^11,17–20^, its role in the pathogenesis of MPN remains elusive. We observed elevated levels of IL-1α and IL-1β in Jak2V617F knock-in mice as well as MPN patients. We hypothesize that IL-1 might play an important role in the initiation/progression of JAK2 mutant MPN/MF.

In this study, we investigate the contribution of IL-1 signaling in the initiation/progression of MPN/MF using conditional IL-1R1 knockout and Jak2V617F knock-in mouse models of MPN. We also examine the role of IL-1 signaling in clonal expansion of Jak2 mutant hematopoietic stem/progenitors. In addition, we test the effects of anti-IL-1R1 antibodies in homozygous Jak2V617F mouse model of MF. Here, we show that IL-1 signaling contributes to clonal expansion and progression of bone marrow fibrosis, and blocking of IL-1R1 with anti-IL-1R1 antibodies reduces the MPN disease burden and ameliorates bone marrow fibrosis in a Jak2V617F mouse model of MPN.

## Results

### IL-1α and IL-1β expression is elevated in human and mouse MPN

**Fig. 1:**
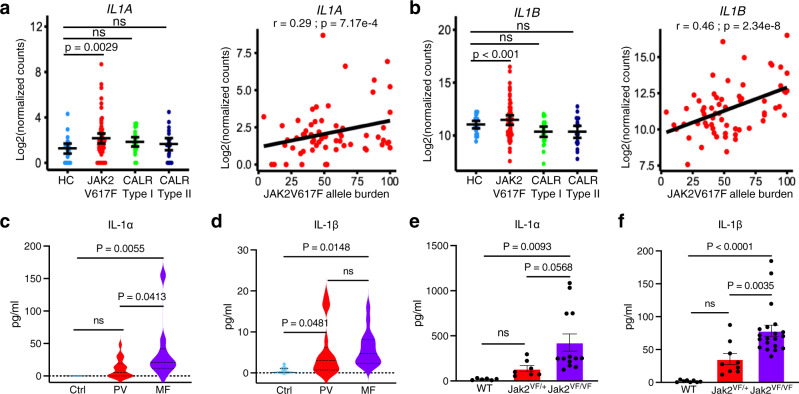
Expression of IL-1 is elevated in MPN. **a** Analysis of publicly available MPN RNA-seq dataset^21^. Left Panel: Expression levels of *IL1A* mRNA in granulocytes of heathy controls (HC) (*n* = 23) and MPN patients with JAK2V617F (*n* = 62), CLAR Type I (*n* = 24) and CLAR Type II (*n* = 22) mutations. Significance was determined using DESeq2 Wald Test. Right Panel: Pearson correlation between *IL1A* mRNA and *JAK2V617F* allele burden in MPN. Two-tailed *t*-test was performed for correlation analysis. **b** Left Panel: Expression levels of *IL1B* mRNA in granulocytes of heathy controls (HC) (*n* = 23) and MPN patients with JAK2V617F (*n* = 62), CLAR Type I (*n* = 24) and CLAR Type II (*n* = 22) mutations. Significance was determined using Wald Test. Right Panel: Pearson correlation between *IL1B* mRNA and *JAK2V617F* allele burden in MPN. Analysis of publicly available MPN RNA-seq dataset^21^. **c** IL-1α level in the plasma of healthy controls (*n* = 11) and patients with PV (*n* = 18) and MF (*n* = 10). **d** IL-1β level in the plasma of healthy controls (*n* = 11) and patients with PV (*n* = 17) and MF (*n* = 16). **e** IL-1α level in the serum of WT control (*n* = 6), heterozygous Jak2V617F (Jak2^VF/+^) (*n* = 7) and homozygous Jak2V617F (Jak2^VF/VF^) (*n* = 13) mice. **f** IL-1β level in the serum of WT (control) (*n* = 8), heterozygous Jak2V617F (Jak2^VF/+^) (*n* = 9) and homozygous Jak2V617F (Jak2^VF/VF^) (*n* = 20) mice. Data are presented as mean ± SEM. Significance was determined in **c**–**f** using one-way ANOVA with Tukey’s multiple comparison test. Source data are provided as a Source Data file.

Analysis of MPN RNA-sequencing data^21^ revealed significantly increased *IL1A (IL-1*α*)* and *IL1B (IL-1β)* mRNA expression in granulocytes of JAK2V617F-positive MPN but not in CALR mutant MPN patients compared to healthy controls (Fig. 1a, b). The mRNA expression of both *IL1A* and *IL1B* positively correlated with JAK2V617F allele burden in granulocytes of MPN patients (Fig. 1a, b). We also assessed the plasma levels of IL-1α and IL-1β in patients with MPN. We observed elevated levels of IL-1α and IL-1β in the plasma of JAK2V617F-positive MPN (PV and MF) patients compared to heathy controls (Fig. 1c, d). Next, we assessed the IL-1α and IL-1β levels in the serum of Jak2V617F knock-in mice^22^. We observed elevated serum IL-1α and IL-1β levels in mice expressing Jak2V617F compared to wild-type (WT) control animals (Fig. 1e, f). Notably, homozygous Jak2V617F (Jak2^VF/VF^) mice exhibited higher levels of IL-1α and IL-1β compared to heterozygous Jak2V617F (Jak2^VF/+^) mice (Fig. 1e, f).

Since MPN/MF are often associated with expansion of myeloid and/or megakaryocytic lineage cells, we assessed the effects of IL-1α and IL-1β on myeloid and megakaryocytic colony outgrowth in WT and Jak2V617F mice bone marrow (BM). We observed significantly increased CFU-GM and CFU-Mk colonies upon ex vivo stimulation of WT and Jak2V617F mice BM with IL-1α or IL-1β (Supplementary Fig. 1a, b). However, the number of CFU-GM and CFU-Mk colonies derived from the BM of Jak2V617F mice were significantly greater compared to WT mice BM both at basal (unstimulated) and IL-1α or IL-1β stimulated conditions (Supplementary Fig. 1a, b). We also observed that stimulation of IL-1α or IL-1β significantly increased in vitro proliferation of primary megakaryocytic cells derived from the BM of WT and Jak2V617F mice (Supplementary Fig. 1c, d).

### Genetic disruption of IL-1 signaling abrogates myelofibrosis in a Jak2V617F mouse model of MF

**Fig. 2:**
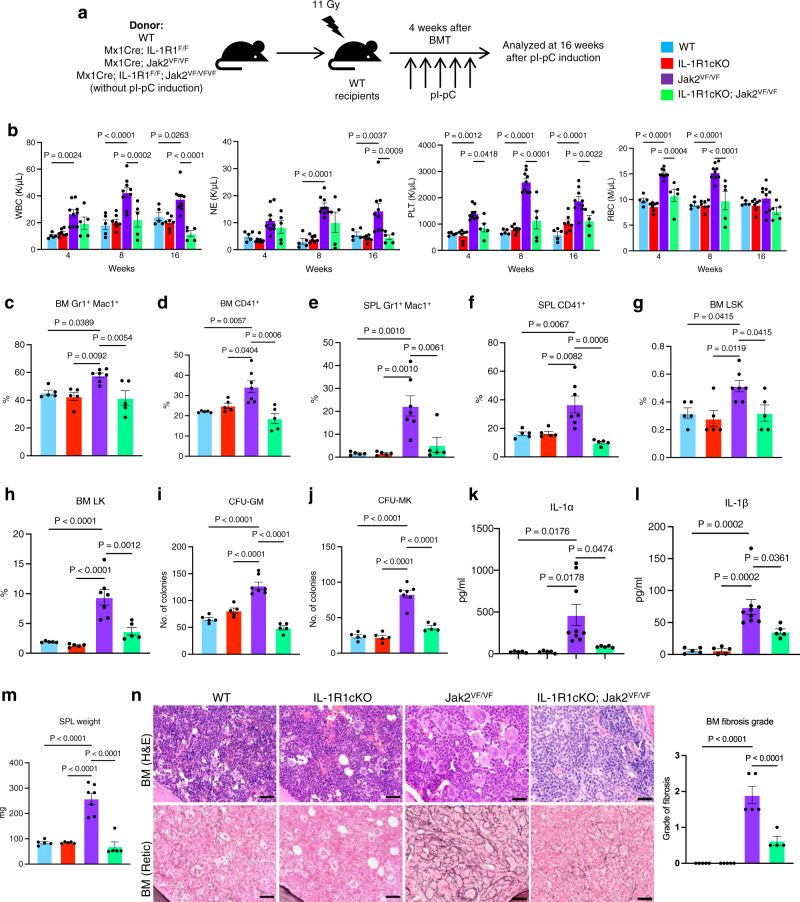
Deletion of IL-1R1 abrogates myelofibrosis in a Jak2V617F mouse model. **a** A scheme on the experimental design is depicted. **b** Peripheral blood white blood cell (WBC), neutrophil (NE), platelet (PLT), and red blood cell (RBC) counts were assessed at 4, 8 and 16 weeks after pI-pC induction in control (WT) (*n* = 5), IL-1R1cKO (*n* = 7), Jak2^VF/VF^ (*n* = 9) and IL-1R1cKO; Jak2^VF/VF^ BMT mice (*n* = 5). Data are presented as mean ± SEM. Significance was determined using two-way ANOVA with Tukey’s multiple comparison test. **c**, **d** Flow cytometric analysis of the frequencies of Gr1^+^/Mac^+^ and CD41^+^ cells in the bone marrow (BM) are shown in bar graphs as mean ± SEM (*n* = 5, 5, 7, 5 mice). **e**, **f** Frequencies of spleen (SPL) Gr1^+^/Mac1^+^ and SPL CD41^+^ cells (*n* = 5, 5, 7, 5). **g**, **h** Frequencies of LSK (**g**) and LK (**h**) cells in the BM of control, IL-1R1cKO, Jak2^VF/VF^ and IL-1R1cKO; Jak2^VF/VF^ mice (*n* = 5, 5, 7, 5). **i** CFU-GM colonies derived from the BM of WT (Control), IL-1R1cKO, Jak2^VF/VF^ and IL-1R1cKO; Jak2 ^VF/VF^ mice (*n* = 5, 5, 7, 5 mice BM samples). **j** CFU-Mk colonies derived from the BM of WT (Control), IL-1R1cKO, Jak2^VF/VF^ and IL-1R1cKO; Jak2 ^VF/VF^ mice (*n* = 5, 5, 7, 5 mice BM samples). **k**, **l** IL-1α and IL-1β levels in the serum of WT, IL-1R1cKO, Jak2^VF/VF^ and IL-1R1cKO; Jak2^VF/VF^ mice (*n* = 5, 5, 9, 5). **m** Spleen weights of WT, IL-1R1cKO, Jak2^VF/VF^ and IL-1R1cKO; Jak2^VF/VF^ mice (*n* = 5, 5, 7, 5). **n** Histopathological analyses of the BM sections stained with H&E and Reticulin staining. Scale bar, 20μm. Histological grade of BM fibrosis (reticulin fibrosis) in WT, IL-1R1cKO, Jak2^VF/VF^ and IL-1R1cKO; Jak2^VF/VF^ mice is shown in bar graphs as mean ± SEM (*n* = 5, 5, 5, 4 mice). Significance was determined in **c**–**n** using one-way ANOVA with Tukey’s multiple comparison test. Source data are provided as a Source Data file.

Next, we sought to determine whether disruption of IL-1 signaling affects the development and progression of myelofibrosis. We previously reported that heterozygous Jak2V617F knock-in mice exhibit a PV disease, whereas mice expressing homozygous Jak2V617F show accelerated development of BM fibrosis^22–24^. So, we utilized homozygous Jak2V617F knock-in (Jak2^VF/VF^) mice to assess the requirement of IL-1 signaling in myelofibrosis development. Since both IL-1α and IL-1β levels were elevated in MPN/MF patients, we deleted the IL-1R1 instead of IL-1α or IL-1β in this study. We crossed Mx1Cre mice^25^ with IL-1R1 floxed^26^ and Jak2V617F knock-in^22^ mice to generate Mx1Cre; IL-1R1^F/F^, Mx1Cre; Jak2^VF/VF^ and Mx1Cre; IL-1R1^F/F^; Jak2^VF/VF^ mice. We transplanted BM cells from control (WT), Mx1Cre; IL-1R1^F/F^ (hereafter IL-1R1cKO), Mx1Cre; Jak2^VF/VF^ (hereafter Jak2^VF/VF^) and Mx1Cre; IL-1R1^F/F^; Jak2^VF/VF^ (hereafter IL-1R1cKO;Jak2^VF/VF^) mice into lethally irradiated C57BL/6 recipient mice to examine the hematopoietic effects of IL-1R1 deletion in a homozygous Jak2V617F setting (as outlined in Fig. 2a). At 4 weeks after transplantation, the recipient animals were injected with pI-pC (polyinosine-polycytosine) to induce Jak2V617F expression as well as IL-1R1 deletion. Mice were analyzed at 16 weeks after pI-pC induction. As expected, mice expressing homozygous Jak2V617F (Jak2^VF/VF^) exhibited a significant increase in white blood cell (WBC), neutrophil (NE) and platelet (PLT) counts compared to WT control mice (Fig. 2b). Conditional deletion of IL-1R1 in the homozygous Jak2V617F mice significantly reduced the WBC, neutrophil and platelet counts to almost wild-type level (Fig. 2b). RBC counts were significantly elevated in Jak2^VF/VF^ mice at early stage but declined at a later stage (Fig. 2b) possibly due to progression towards bone marrow fibrosis. Deletion of IL-1R1 in the homozygous Jak2V617F mice normalized RBC counts and they were comparable to that seen in WT control animals (Fig. 2b). Mice with IL-1R1 deletion alone (IL-1R1cKO) did not exhibit any significant alterations in peripheral blood WBC, neutrophil, platelet and RBC counts compared with WT control animals (Fig. 2b). Flow cytometric analysis showed a significant expansion of Gr-1^+^ Mac1^+^ myeloid and CD41^+^ megakaryocyte-lineage cells in the BM and spleens of Jak2^VF/VF^ mice (Fig. 2c–f). The CD41^+^ compartment includes megakaryocytic cells and their progenitors, and stem cell-like megakaryocyte committed progenitors^27,28^. Deletion of IL-1R1 caused significant decrease of Gr-1^+^ Mac1^+^ and CD41^+^ cells in the BM and spleens of IL-1R1cKO; Jak2^VF/VF^ mice compared to Jak2^VF/VF^ mice (Fig. 2c–f).

We next assessed the effects of IL-1R1 deletion on Jak2^VF/VF^ mice hematopoietic stem/progenitor cells (HSPC). Jak2^VF/VF^ mice exhibited significant increase of LSK (Lin^-^Sca1^+^c-kit^+^) and myeloid progenitors (LK; Lin^-^c-kit^+^) in their BM (Fig. 2g, h). Deletion of IL-1R1 significantly reduced the LSK and myeloid progenitors in the BM of IL-1R1cKO; Jak2^VF/VF^ mice compared to Jak2^VF/VF^ mice (Fig. 2g, h). Hematopoietic progenitor colony assays showed that the number of CFU-GM and CFU-Mk colonies derived from the BM of Jak2^VF/VF^ mice were significantly greater compared to WT control animals (Fig. 2i, j). The number of CFU-GM and CFU-Mk colonies derived from the BM of IL-1R1cKO; Jak2^VF/VF^ mice were significantly lower compared to Jak2^VF/VF^ mice (Fig. 2i, j). Mice with only IL-1R1 deletion (IL-1R1cKO), however, did not exhibit any significant alterations in LSK/progenitors and myeloid/megakaryocytic colony formation as compared to WT control mice (Fig. 2g–j). Interestingly, IL-1R1cKO; Jak2^VF/VF^ mice displayed significantly reduced serum IL-1α and IL-1β levels compared to Jak2^VF/VF^ mice (Fig. 2k, l). Spleen weight was significantly reduced in IL-1R1cKO; Jak2^VF/VF^ mice compared with Jak2^VF/VF^ mice and they were comparable to control WT mice (Fig. 2m). H&E and reticulin staining of the BM sections from Jak2^VF/VF^ mice showed increased clusters of atypical megakaryocytes and extensive BM fibrosis, whereas IL-1R1cKO; Jak2^VF/VF^ mice exhibited reduced megakaryocytes and significant decrease of bone marrow fibrosis (Fig. 2n). BM histology from IL-1R1cKO mice were comparable to that observed in WT control animals (Fig. 2n). Overall, these data suggest a role for IL-1 signaling in Jak2V617F-evoked myelofibrosis.

### Disruption of IL-1 signaling inhibits clonal expansion of Jak2V617F mutant HSPC

**Fig. 3:**
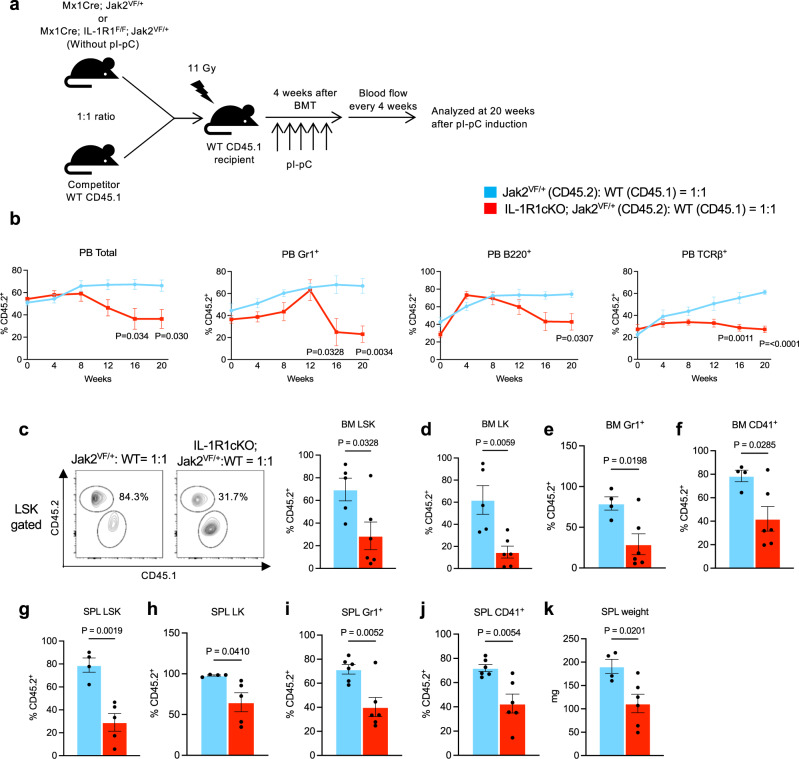
Deletion of IL-1R1 preferentially inhibits Jak2V617F mutant hematopoietic stem/progenitor cells. **a** A scheme on competitive BM transplantation approach to assess the effects of IL-1R1 deletion on Jak2V617F mutant hematopoietic stem/progenitors is depicted. **b** Percentages of donor-derived (CD45.2^+^) total, myeloid cells (Gr1^+^), B cells (B220^+^), and T cells (TCRβ^+^) in peripheral blood were measured every 4 weeks after pI-pC injection (*n* = 5, 4, 4, 4, 4, 4 recipient mice for Jak2^VF/+^ donor, *n* = 7, 7, 7, 6, 6, 6 mice for IL-1R1cKO; Jak2^VF/+^ donor at 0, 4, 8, 12, 16 and 20 weeks, respectively). Statistical significance was determined using multiple unpaired two-tailed *t*-tests. **c** Representative flow cytometric plots on the ratio of CD45.2^+^ vs. CD45.1^+^ LSK cells in the BM of recipient mice are shown on the left, and the percentages of donor-derived (CD45.2^+^) LSK in the BM of recipient mice (*n* = 5, 6) are shown in bar graphs as mean ± SEM on the right. **d**–**f** Percentages of donor-derived (CD45.2^+^) LK (**d**), Gr1^+^ (**e**) and CD41^+^ (**f**) cells in the BM of recipient animals at 20 weeks after pI-pC injection are shown as mean ± SEM (*n* = 4, 6). **g**–**j** Percentages of donor-derived (CD45.2^+^) LSK (*n* = 4, 5) (**g**), LK (*n* = 4, 5) (**h**), Gr1^+^ (*n* = 6, 6) (**i**) and CD41^+^ (*n* = 6, 6) (**j**) cells in the spleens of recipient animals are shown as mean ± SEM. **k** Spleen weights in the recipient animals of Jak2^VF/+^ and IL-1R1cKO; Jak2^VF/+^ mice BM (*n* = 4, 6). Statistical significances in **c**–**k** were determined using two-tailed unpaired *t*-test. Source data are provided as a Source Data file.

Next, we asked whether disruption of IL-1 signaling could inhibit the expansion of JAK2 mutant HSPC. To address this question, we performed competitive transplantation assays using BM from Mx1Cre; Jak2^VF/+^ (inducible heterozygous Jak2V617F) and Mx1Cre; IL-1R1^F/F^; Jak2^VF/+^ (inducible IL-1R1 knockout heterozygous Jak2V617F) mice. BM cells (5 × 10^5^) from uninduced Mx1Cre; Jak2^VF/+^ or Mx1Cre; IL-1R1^F/F^; Jak2^VF/+^ mice (CD45.2^+^) were mixed with CD45.1^+^ WT mice BM cells (5 × 10^5^) at a ratio of 1:1 and then transplanted into lethally irradiated CD45.1^+^ congenic recipient animals (as outlined in Fig. 3a). At 4 weeks after BMT, the recipient animals were injected with pI-pC to induce Jak2V617F expression and IL-1R1 deletion. The percentages of donor-derived (CD45.2^+^) cells were determined in the peripheral blood leukocytes of the chimeric mice by flow cytometry every 4 weeks and the mice were analyzed at 20 weeks after pI-pC induction. We observed significantly higher percentages of total CD45.2^+^ cells as well as CD45.2^+^ myeloid (Gr-1^+^), B- and T-cells in the peripheral blood of chimeric mice receiving Jak2^VF/+^ BM compared with chimeric mice receiving IL-1R1cKO; Jak2^VF/+^ BM (Fig. 3b). Whereas the percentages of CD45.2^+^ cells derived from Jak2^VF/+^ donor BM were expanded overtime, percentages of CD45.2^+^ cells originated from IL-1R1cKO; Jak2^VF/+^ BM were rather decreased in the peripheral blood of chimeric recipient animals (Fig. 3b). We also observed significantly reduced percentages of CD45.2^+^ LSK, LK, Gr-1^+^ and CD41^+^ cells in the BM of chimeric recipient animals receiving IL-1R1cKO; Jak2^VF/+^ BM compared with Jak2^VF/+^ BM (Fig. 3c–f). Similarly, we observed significantly reduced percentages of CD45.2^+^ LSK, LK, Gr-1^+^ and CD41^+^ cells in the spleens of chimeric animals receiving IL-1R1cKO; Jak2^VF/+^ BM compared with Jak2^VF/+^ BM (Fig. 3g–j). We also observed a significant decrease in spleen weight in recipients of IL-1R1cKO; Jak2^VF/+^ BM compared with Jak2^VF/+^ BM (Fig. 3k). Together, these results suggest a significant role for IL-1 signaling in clonal expansion of Jak2V617F mutant HSPC.

### IL-1 signaling in mesenchymal stromal cells is required for efficient induction of bone marrow fibrosis in Jak2V617F mice

**Fig. 4:**
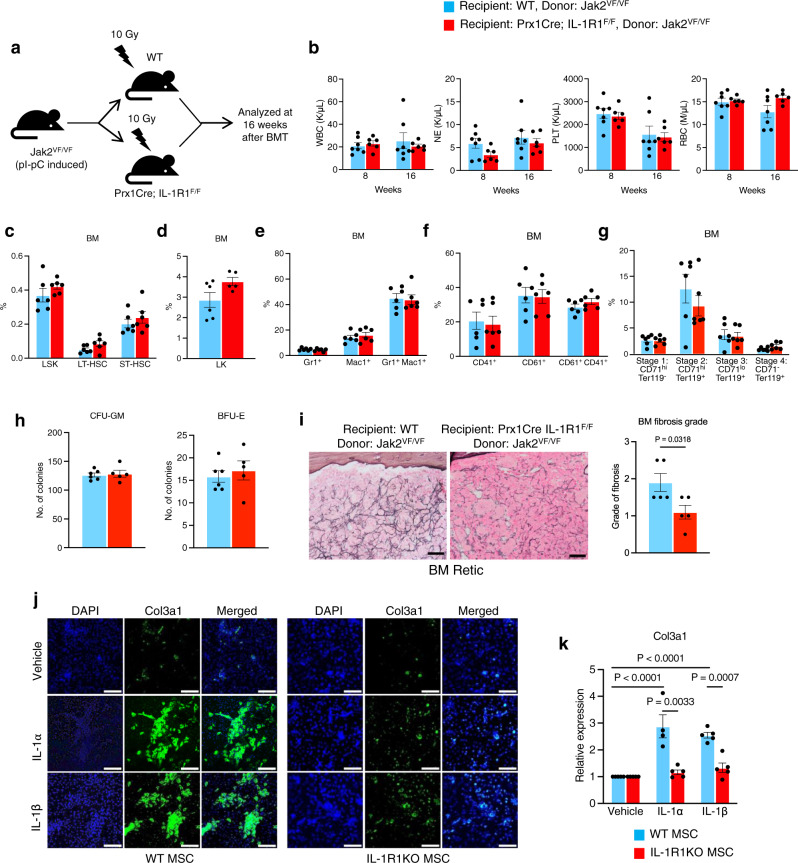
Effects of IL-1R1 deletion in BM MSC on Jak2V617F-induced MPN. **a** A scheme on the experimental design is depicted. **b** Peripheral blood WBC, NE, PLT, and RBC counts at 8 and 16 weeks after transplantation in WT (*n* = 7) and Prx1Cre; IL-1R1^F/F^ recipient mice (*n* = 6). Data are shown as mean ± SEM. Statistical significance was determined using multiple unpaired two-tailed *t*-tests. **c** Frequencies of LSK (Lin^−^ Sca1^+^ c-kit^+^), LT-HSC (Lin^-^ Sca1^+^ c-kit^+^ CD34^-^ CD135^-^) ST-HSC (Lin^-^ Sca1^+^ c-kit^+^ CD34^+^ CD135^-^) and LK (Lin^-^c-kit^+^) in the BM of WT or Prx1Cre; IL-1R1^F/F^ recipients at 16 weeks after transplantation (*n* = 6, 6). All bar graphs represent mean ± SEM. **d** Frequencies of LK (Lin^-^c-kit^+^) in the BM of WT or Prx1Cre; IL-1R1^F/F^ recipients (*n* = 6, 5). **e**–**g** Percentages of Gr1^+^/Mac1^+^ (**e**), CD61^+^/CD41^+^ (**f**) and CD71/Ter119 (**g**) cells in the BM of WT and Prx1Cre; IL-1R1^F/F^ recipients (*n* = 6, 6 mice). **h** CFU-GM and BFU-E colonies in the BM of WT and Prx1Cre; IL-1R1^F/F^ recipients (*n* = 6, 5 mice). **i** Representative images of the reticulin staining of the BM sections from recipient mice at 16 weeks after transplantation. Scale bar, 20 μm. Histological grade of BM fibrosis in WT and Prx1Cre; IL-1R1^F/F^ recipients is shown in bar graphs as mean ± SEM (*n* = 5 mice per group). Statistical significances were determined using two-tailed unpaired *t*-test. **j** Immunofluorescence images showing Col3a1 expression in WT and IL-1R1 KO BM MSCs upon IL-1α (5 ng/mL) and IL-1β (5 ng/mL) stimulation. Col3a1 (green) and DAPI (blue); scale bars, 100 µm. Representative images from 3 independent experiments are shown. **k** WT and IL-1R1 KO mice BM MSCs were treated with vehicle (PBS), IL-1α (5 ng/mL) or IL-1β (5 ng/mL) for 72 h. Col3a1 mRNA expression was determined by RT-qPCR. Fold change of Col3a1 expression is shown in bar graphs as mean ± SEM (*n* = 5, 4, 5 biological replicates for vehicle, IL-1α and IL-1β treatment in WT MSCs; *n* = 5, 5, 5 biological replicates for vehicle, IL-1α and IL-1β treatment in IL-1R1 KO MSCs). Statistical significance was determined in **k** using two-way ANOVA with Tukey’s multiple comparison test. Source data are provided as a Source Data file.

Previous studies have suggested that disruption of IL-1 signaling does not have significant impact on normal hematopoietic development^12,13,26^. Analysis of RNA-sequencing and microarray gene expression data^29–31^ revealed that IL1B and IL1R1 are expressed in HSC, megakaryocytes, neutrophils, monocytes and dendritic cells (Supplementary Fig. 2). IL1R1 is highly expressed in the bone marrow stromal cells (Supplementary Fig. 2). In order to address the role of IL-1 signaling in BM microenvironment and its contribution in the pathogenesis of Jak2V617F-induced myelofibrosis, we crossed IL-1R1 floxed mice^26^ with Prx1Cre transgenic mice^32^ to generate Prx1Cre; IL-1R1^F/F^ mice that allow Cre-mediated deletion of IL-1R1 in BM mesenchymal stromal cells (MSC). BM cells (1 × 10^6^) from pI-pC induced homozygous Jak2V617F (Jak2^VF/VF^) mice were transplanted into lethally irradiated WT and Prx1Cre; IL-1R1^F/F^ C57BL/6 recipient mice and analyzed at 16 weeks after transplantation (as outlined in Fig. 4a). We observed no significant changes in peripheral blood WBC, neutrophil (NE), platelet (PLT) and RBC counts between WT and Prx1Cre; IL-1R1^F/F^ recipient animals transplanted with Jak2^VF/VF^ BM cells (Fig. 4b). We also examined the effects of IL-1R1 deletion in BM mesenchymal stromal cells on HSPC. The gating strategy for HSPC analysis is shown in Supplementary Fig. 3. Flow cytometric analyses showed no significant changes in the LSK, LT-HSC, ST-HSC and LK populations in the BM and spleens between Prx1Cre; IL-1R1^F/F^ and WT recipients of Jak2^VF/VF^ BM cells (Fig. 4c, d and Supplementary Fig. 4a, b). We also did not observe any significant differences in myeloid (Gr1^+^/Mac1^+^), erythroid (CD71^+^/Ter119^+^) and megakaryocytic (CD41^+^/CD61^+^) lineage cells in the BM and spleens between Prx1Cre; IL-1R1^F/F^ and WT recipients of Jak2^VF/VF^ BM cells (Fig. 4e–g and Supplementary Fig. 4c–e). Hematopoietic progenitor colony assays showed no significant differences in CFU-GM and BFU-E colonies between Prx1Cre; IL-1R1^F/F^ and WT recipients of Jak2^VF/VF^ BM cells (Fig. 4h). Bone marrow histologic analyses revealed extensive BM fibrosis in WT recipients of Jak2^VF/VF^ mice BM at 16 weeks after transplantation (Fig. 4i). Prx1Cre; IL-1R1^F/F^ recipients exhibited a significant reduction of BM fibrosis compared to WT recipients (Fig. 4i). These data suggest that IL-1 signaling in mesenchymal stromal cells is required for efficient induction of BM fibrosis.

To further examine the contribution of IL-1 signaling in BM fibrosis, we directly assessed the effects of IL-1α and IL-1β stimulation on collagen expression in mesenchymal stromal cells (MSC) derived from WT and Prx1Cre; IL-1R1^F/F^ (IL-1R1 KO) mice BM. Confocal microscopy and real-time qPCR analyses showed significantly increased collagen (Col3a1) expression in WT BM MSC following IL-1α or IL-1β stimulation (Fig. 4j, k). Prx1Cre-mediated deletion of IL-1R1 significantly reduced collagen (Col3a1) expression in BM MSC following IL-1α or IL-1β stimulation (Fig. 4j, k). These results establish a role for IL-1 signaling in BM matrix deposition and fibrosis.

Next, we asked whether disruption of IL-1 signaling in BM stromal cells could inhibit the expansion of JAK2 mutant stem/progenitor cells. To address this question, BM cells (5 × 10^5^) from pI-pC induced Mx1Cre; Jak2^VF/+^; GFP^+^ mice (referred as Jak2^VF/+^; GFP^+^) were mixed with C57BL/6 WT mice BM cells (5 × 10^5^) at a ratio of 1:1 and transplanted into lethally irradiated WT or Prx1Cre; IL-1R1^F/F^ C57BL/6 recipient mice (as outlined in Supplementary Fig. 5a). Recipient animals were analyzed at 20 weeks after transplantation. We did not observe any significant changes in peripheral blood WBC, neutrophil, platelet and RBC counts between WT and Prx1Cre; IL-1R1^F/F^ recipient animals (Supplementary Fig. 5b). Also, there were no significant differences in the percentages of total GFP^+^ (Jak2 mutant) cells as well as GFP^+^ Gr1^+^, CD41^+^, Ter119^+^, TCRβ^+^ and B220^+^ cells in the peripheral blood between WT and Prx1Cre; IL-1R1^F/F^ recipient animals (Supplementary Fig. 5c). The percentages of GFP^+^ (Jak2 mutant) LSK, LT-HSC and ST-HSC in the BM and spleens between Prx1Cre; IL-1R1^F/F^ and WT recipient animals were comparable (Supplementary Fig. 5d, e). We also did not observe significant differences in GFP^+^ Gr1^+^, CD41^+^, Ter119^+^, B220^+^ and TCRβ^+^ cells in the BM and spleens between WT and Prx1Cre; IL-1R1^F/F^ recipient animals (Supplementary Fig. 5f, g). These results suggest that disruption of IL-1 signaling in the BM microenvironment has no significant effect on expansion of Jak2 mutant HSPC.

### Exogenous IL-1β promotes MPN disease progression in Jak2V617F mice

**Fig. 5:**
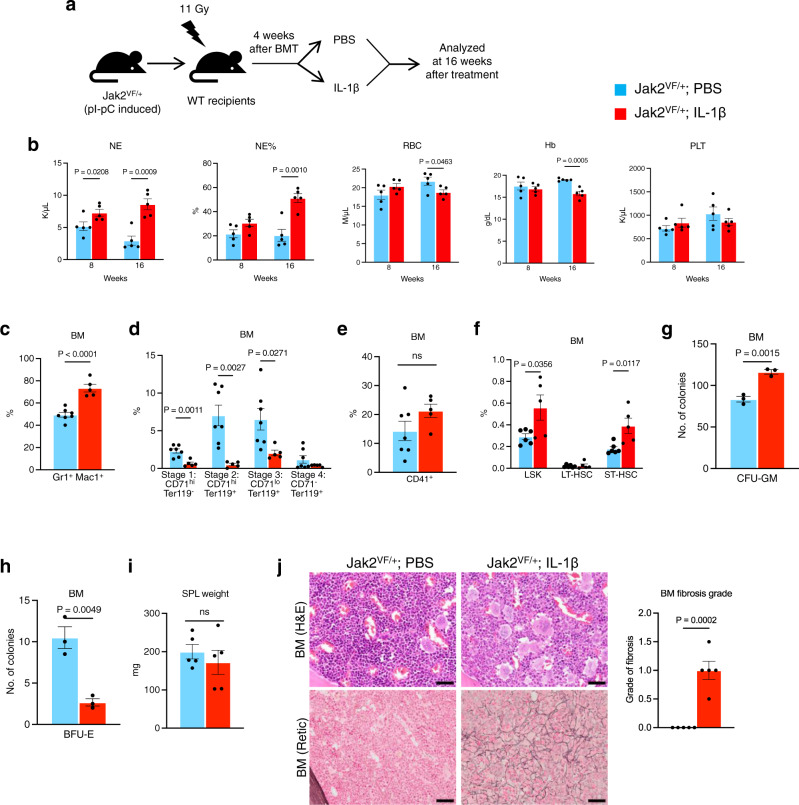
Exogenous IL-1 treatment promotes the development of bone marrow fibrosis in Jak2V617F mice. **a** A scheme on the experimental design is depicted. **b** Peripheral blood counts of neutrophil (NE), percentages of neutrophil (%NE), red blood cells (RBC), hemoglobin (Hb) and platelets (PLT) were assessed at 8 and 16 weeks after treatment (*n* = 5 mice per group). **c**–**e** Frequencies of Gr1^+^Mac1^+^, CD71^+^/Ter119^+^ and CD41^+^ cells in the BM of Jak2^VF+^ mice treated with PBS or IL-1β are shown in bar graphs as mean ± SEM (*n* = 7, 5 mice). **f** Frequencies of LSK (Lin^−^ Sca1^+^ c-kit^+^), LT-HSC (Lin^-^ Sca1^+^ c-kit^+^ CD34^-^ CD135^-^) and ST-HSC (Lin^-^ Sca1^+^ c-kit^+^ CD34^+^ CD135^-^) in the BM of Jak2^VF+^ mice treated with PBS or IL-1β are shown in bar graphs as mean ± SEM (*n* = 6, 5 mice). **g**, **h** BM cells (2 × 10^4^) from PBS or IL-1β treated Jak2^VF+^ mice were plated in methylcellulose medium (MethoCult 3434) with cytokines. Colony forming unit granulocyte-macrophage (CFU-GM) (**g**) and burst forming unit erythroid (BFU-E) (**h**) colonies are shown in bar graphs as mean ± SEM (*n* = 3 mice per group; each data point is an average of two technical replicates). **i** Spleen weights of Jak2^VF+^ mice treated with PBS and IL-1β (*n* = 5 mice per group). **j** Representative images of the hematoxylin and eosin (H&E) and reticulin stained BM sections from Jak2^VF+^ mice treated with PBS or IL-1β for 16 weeks. Scale bar, 20 μm. Histological grade of BM fibrosis (reticulin fibrosis) is shown in bar graphs as mean ± SEM (*n* = 5 mice per group). Statistical significances were determined in **b**–**j** using two-tailed unpaired t-test. Source data are provided as a Source Data file.

In order to examine the effect of increased IL-1 signaling in MPN progression, we performed exogenous IL-1β administration in heterozygous Jak2V617F (Jak2^VF/+^) knock-in mice that mainly exhibit a PV disease^22^. BM cells from pI-pC induced Jak2^VF/+^ mice were transplanted into lethally irradiated C57BL/6 mice to generate a cohort of mice expressing Jak2^VF/+^. At 4 weeks after transplantation, mice were injected with either PBS (control) or IL-1β (0.5 μg/dose, intraperitoneally) 3 times per week for 16 weeks (as outlined in Fig. 5a). Treatment of IL-1β significantly increased the numbers and percentages of neutrophils but decreased the RBC and hemoglobin levels in the peripheral blood of Jak2^VF/+^ mice (Fig. 5b). Platelet counts were not significantly altered by IL-1β treatment in these mice (Fig. 5b). Flow cytometric analyses showed significantly increased myeloid (Gr-1^+^ Mac1^+^) cells and decreased percentages of erythroid (CD71^+^/Ter119^+^) cells but no significant alterations of CD41^+^ cells in the BM of IL-1β treated Jak2^VF/+^ mice compared to PBS treated animals (Fig. 5c–e). We also observed significant increases in LSK and short-term HSC (ST-HSC) in Jak2^VF/+^ mice treated with IL-1β compared to those treated with PBS (Fig. 5f). Hematopoietic progenitor colony assays showed significantly increased number of CFU-GM (myeloid) but decreased BFU-E (erythroid) colonies derived from the BM of IL-1β treated mice compared with PBS treated animals (Fig. 5g, h). However, spleen size was not significantly altered by IL-1β treatment in these animals (Fig. 5i). H&E staining of the BM sections from PBS treated Jak2^VF/+^ mice exhibited trilineage hyperplasia with noticeable increase in erythroid precursors, whereas IL-1β treated Jak2^VF/+^ mice showed expansion of granulocytic cells and immature megakaryocytes and reduction of erythroid precursors (Fig. 5j). While PBS treated Jak2^VF/+^ mice did not exhibit BM fibrosis at this stage, IL-1β treated mice exhibited increased reticulin fibrosis in their BM (Fig. 5j). Together, these results suggest that elevated levels of IL-1β may contribute to the progression of Jak2V617F-induced MPN.

We also examined the effect of exogenous IL-1β in WT mice. C57BL/6 WT mice were injected with either PBS (control) or IL-1β intraperitoneally 3 times per week for 16 weeks (outlined in Supplementary Fig. 6a). Mice treated with IL-1β exhibited significantly increased WBC and neutrophil counts in their peripheral blood compared to PBS treatment (Supplementary Fig. 6b). RBC, hemoglobin and platelet levels were comparable between PBS and IL-1β treated WT mice (Supplementary Fig. 6b). Flow cytometric analyses showed significantly increased myeloid (Gr-1^+^ Mac1^+^) cells and decreased percentage of erythroid (CD71^+^/Ter119^+^) cells but no significant alterations of CD41^+^ cells in the BM of IL-1β treated WT mice compared to PBS treated animals (Supplementary Fig. 6c–e). There was no significant difference in LSK, LT-HSC and ST-HSC populations between PBS and IL-1β treated WT mice (Supplementary Fig. 6f). Hematopoietic progenitor colony assays showed significantly increased CFU-GM (myeloid) but decreased BFU-E (erythroid) colonies in the BM of IL-1β treated mice compared with PBS treated animals (Supplementary Fig. 6g, h). Spleen weights were comparable between PBS and IL-1β treated WT mice (Supplementary Fig. 6i). Histologic analysis of the BM sections showed increase in granulocytic precursors without any evidence of BM fibrosis in WT mice treated IL-1β (Supplementary Fig. 6j). Thus, IL-1β treatment alone cannot induce BM fibrosis in a WT background.

### Effects of IL-1β on gene expression in Jak2V617F mice hematopoietic progenitors

**Fig. 6:**
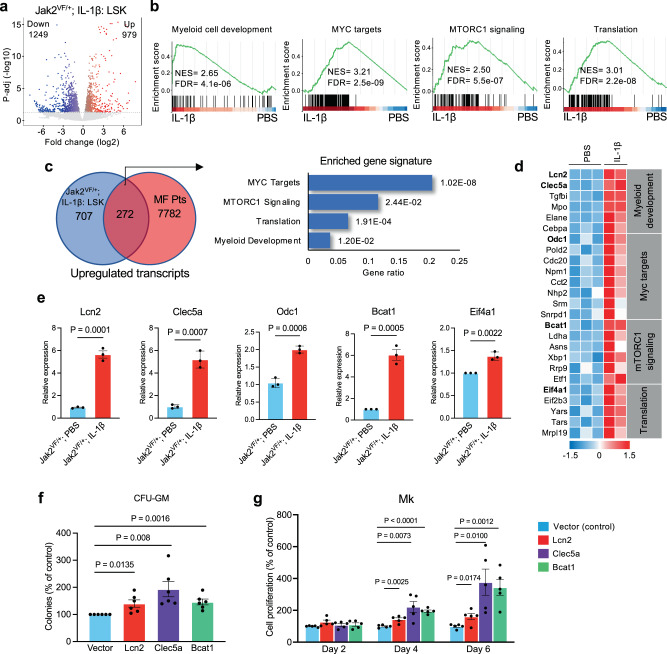
Effects of IL-1β on gene expression in Jak2V617F mice hematopoietic progenitors. **a** Volcano plot showing significantly upregulated and downregulated (p-adj < 0.05 and log_2_fc > 0.5) genes in in LSK cells isolated from Jak2^VF/+^ mice treated with vehicle (PBS) (*n* = 3) or IL-1β (*n* = 2). **b** Gene-set enrichment analysis (GSEA). Gene sets of myeloid cell development, MYC targets, mTORC1 signaling and translation are enriched in LSK cells from Jak2^VF/+^ mice treated with IL-1β (*n* = 2) compared to PBS (*n* = 3). **c** Venn diagram showing the overlap between upregulated genes in MF patient granulocytes^21^ (*n* = 62 for MF, *n* = 23 for control) and genes upregulated in IL-1β treated Jak2^VF/+^ LSK cells (*n* = 2 for IL-1β treated, *n* = 3 for PBS treated). The cutoffs were FDR-adjusted *p* < 0.05. Overlapping genes showed enrichment for MYC targets, mTORC1 signaling, translation and myeloid development gene signatures. **d** Heat maps of selected upregulated transcripts related to MYC targets, mTORC1 signaling, translation and myeloid development gene signatures in LSK cells from Jak2^VF/+^ mice treated with IL-1β (*n* = 2) compared to PBS (*n* = 3) (FDR < 0.05). Gene transcripts shown in bold were further validated. **e** RT-qPCR validation of Lcn2, Clec5a, Odc1, Bcat1 and Eif4a1 mRNA expression in LSK cells from Jak2^VF/+^ mice treated with IL-1β compared to PBS. RT-qPCR results were normalized with Hprt1 expression (*n* = 3 biological replicates per group). **f** CFU-GM colonies were assessed following overexpression of Lcn2, Clec5a and Bcat1 in Jak2^VF/+^ BM. CFU-GM colonies relative to vector control are shown in bar graphs as mean ± SEM (*n* = 6 biological replicates per group; each data point is an average of two technical replicates). **g** Megakaryocytic (Mk) cells were derived from the Jak2^VF/+^ BM overexpressing vector, Lcn2, Clec5a and Bcat1, and cell proliferation was assessed in triplicates every 2 days over 6 days using Cell Titer Glow. Megakaryocytic cell proliferation relative to vector control are shown in bar graphs as mean ± SEM (*n* = 5 biological replicates per group; each data point is an average of two technical replicates). Statistical significances were determined in **e**–**g** using multiple unpaired two-tailed t-tests. Source data are provided as a Source Data file.

To understand the mechanism(s) by which elevated IL-1β expression contributes to myeloid expansion and progression of MPN, we performed RNA-sequencing on sorted LSK and LK cells from Mx1Cre; Jak2^VF/+^ mice treated with vehicle (PBS) and IL-1β. Since IL-1β contributes to expansion of HSPC in Jak2V617F mice, we utilized LSK and LK cells for transcriptomic analysis. We found that 979 transcripts were significantly (p-adj < 0.05 and log2FC > 0.5-fold) upregulated and 1249 transcripts were significantly down-regulated in IL-1β-treated LSK cells compared to vehicle-treated LSK cells (Fig. 6a). Gene Set Enrichment Analysis (GSEA)^33^ of RNA-sequencing data revealed significant upregulation of genes related to myeloid cell development, MYC targets, mTORC1 signaling and translation in IL-1β -treated Jak2^VF/+^ mice LSK cells compared to vehicle-treated LSK cells (Fig. 6b). RNA-seq analysis on LK cells from IL-1β-treated Jak2^VF/+^ mice also showed significant changes in gene expression (Supplementary Fig. 7a). GSEA analyses showed significant upregulation of genes related to myeloid leukocyte activation, innate immune system, cytokine production and bone marrow neutrophil gene signatures in IL-1β-treated Jak2^VF/+^ mice LK cells compared to vehicle-treated LK cells (Supplementary Fig. 7b). Analysis of the RNA-seq data from IL-1β -treated WT mice multipotent progenitors (MPP)^34^ also showed significant upregulation of genes related to mTORC1 signaling, translation, inflammatory response and myeloid cell development (Supplementary Fig. 8a, b). We observed 266 gene transcripts with significant overlap between IL-1β -treated WT mice MPP and Jak2^VF/+^ mice LSK cells (Supplementary Fig. 8c).

We next compared genes/transcripts that were significantly upregulated in IL-1β-treated Jak2^VF/+^ mice LSK cells with transcripts that were upregulated in MF patient’s RNA-sequencing dataset^21^. There was an overlap of 272 gene transcripts, which were upregulated in both MF patient’s granulocytes and IL-1β-treated Jak2^VF/+^ mice LSK cells (Fig. 6c). Gene Ontology analysis of overlapping genes showed enrichment for MYC targets, mTORC1 signaling, translation and myeloid development gene signatures (Fig. 6c), indicating that these pathways were commonly upregulated in both MF patient’s hematopoietic cells and IL-1β-treated Jak2^VF/+^ mice LSK cells. We also compared the RNA-seq data obtained from IL-1β-treated Jak2^VF/+^ mice LSK cells with MF CD34 + microarray gene expression dataset^35^ and found similar enrichment for MYC targets, mTORC1 signaling, translation and myeloid development gene signatures (Supplementary Fig. 9a). There was also significant overlap of upregulated genes between IL-1β-treated Jak2^VF/+^ mice LK cells and MF patient’s granulocytes (Supplementary Fig. 7c). Thus, increased level of IL-1β alters the expression of genes that may contribute to expansion of myeloid cells in JAK2 mutant MPN.

We found that transcripts related to myeloid development, MYC targets, mTORC1 signaling and translation, which include Lcn2, Clec5a, Odc1, Bcat1 and Eif4a1, were significantly upregulated in IL-1β-treated Jak2^VF/+^ mice LSK cells by RNA-seq analysis (Fig. 6d). Expression of these transcripts was also found significantly upregulated in MF patient’s granulocytes (Supplementary Table 1). RT-qPCR further validated significantly increased expression of these transcripts in IL-1β-treated Jak2^VF/+^ mice LSK cells compared with PBS-treated Jak2^VF/+^ mice LSK cells (Fig. 6e). Interestingly, the expression of *LCN2, CLEC5A, ODC1, BCAT1* and *EIF4A1* was also significantly elevated in MF CD34 + cells (Supplementary Fig. 9b).

We further performed functional validation of some of these IL-1β target genes by lentiviral overexpression into Jak2^VF/+^ mice BM. We observed significantly increased myeloid (CFU-GM) colonies by overexpression of Lcn2, Clec5a or Bcat1 in the BM of Jak2^VF/+^ mice (Fig. 6f). We also observed that overexpression of Lcn2, Clec5a or Bcat1 significantly increased ex vivo proliferation of megakaryocytes derived from the BM of Jak2^VF/+^ mice (Fig. 6g).

### Effects of IL-1 on gene expression changes and collagen expression in BM mesenchymal stromal cells

**Fig. 7:**
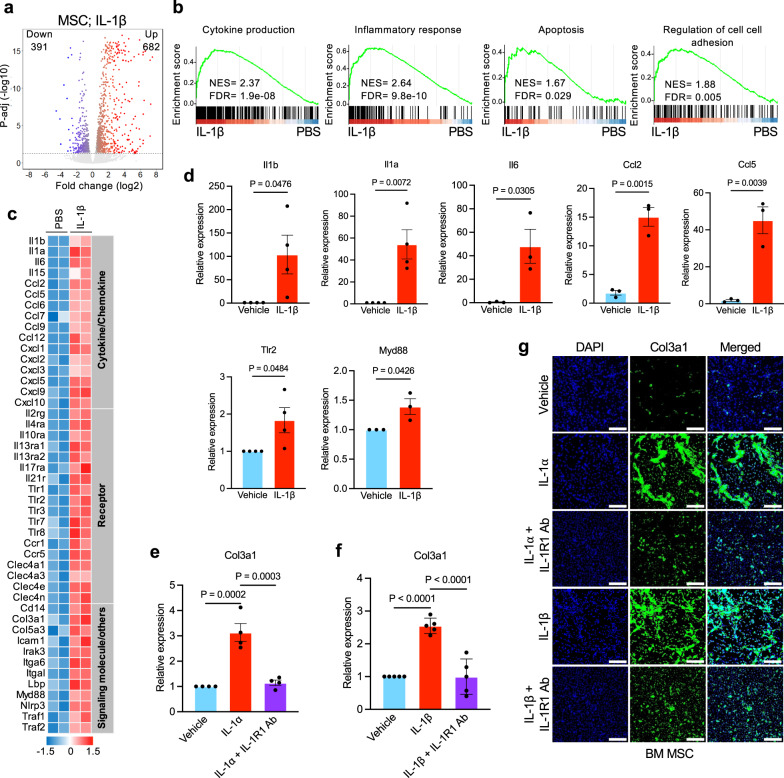
Effects of IL-1 on gene expression changes and collagen expression in BM mesenchymal stromal cells. **a** Volcano plot showing significantly upregulated and downregulated (p-adj < 0.05 and log_2_fc > 0.5) genes in IL-1β (5 ng/mL) treated MSCs (*n* = 2) compared to PBS treated MSCs (*n* = 2). **b** Gene-set enrichment analyses (GSEA) show significant increase in expression of genes related to cytokine production, inflammatory response, apoptosis and regulation of cell-cell adhesion in IL-1β treated MSCs (*n* = 2) compared to PBS treated MSCs (*n* = 2). Enrichment plots with normalized enrichment score (NES) and false discovery rate (FDR) are shown. **c** Heat map of selected genes upregulated in IL-1β treated MSCs (*n* = 2) compared to PBS treated MSCs (*n* = 2) with expression changes more than 1.5-fold (FDR < 0.05). **d** RT-qPCR validation of increased mRNA expression of Il1b (*n* = 4, 4 biological replicates), Il1a (*n* = 4, 4), Il6 (*n* = 3, 3), Ccl2 (*n* = 3, 3), Ccl5 (*n* = 3, 3), Tlr2 (*n* = 4, 4) and Myd88 (*n* = 3, 3) in IL-1β treated MSCs compared with PBS treated MSCs. Data were normalized with Hprt1 expression. Data are shown in bar graphs as mean ± SEM. Statistical significances were determined using two-tailed unpaired *t*-test. **e**, **f** BM MSCs were treated with vehicle (PBS), IL-1α (5 ng/mL) or IL-1α (5 ng/mL) + IL-1R1 Ab (1 μg/ml) (*n* = 4 biological replicates per group) (**e**) and vehicle (PBS), IL-1β (5 ng/mL) or IL-1β (5 ng/mL) + IL-1R1 Ab (1 μg/ml) (*n* = 5 biological replicates per group) (**f**) for 72 h. Col3a1 mRNA expression was assessed using RT-qPCR. Fold change of Col3a1 expression is shown in bar graphs as mean ± SEM. Statistical significance was determined using one-way ANOVA with Tukey’s multiple comparison test. **g** Immunofluorescence images showing increased Col3a1 expression in MSCs upon IL-1β (5 ng/mL) and IL-1α (5 ng/mL) stimulation and, IL-1R1 Ab (1 μg/ml) treatment abolished IL-1α/β-induced Col3a1 expression in the BM MSCs. Col3a1 (green) and DAPI (blue); scale bars, 100 µm. Representative images from 3 independent experiments are shown. Source data are provided as a Source Data file.

To understand the contribution of IL-1 signaling in BM microenvironment, we investigated the effects of IL-1β on gene expression changes in BM MSC. RNA-seq analysis on BM MSC stimulated with IL-1β showed 682 transcripts were significantly upregulated and 391 transcripts were significantly down-regulated in IL-1β -treated MSC compared to vehicle-treated MSC (Fig. 7a). GSEA analysis revealed significant upregulation of the transcripts related to cytokine production, inflammatory response, apoptosis and regulation of cell-cell adhesion in IL-1β-treated MSC compared to vehicle-treated MSC (Fig. 7b). Transcripts encoding cytokines, chemokines and signaling molecules with known proinflammatory functions were upregulated in IL-1β-treated MSC (Fig. 7c). Consistent with this observation, RT-qPCR analysis revealed that IL-1β treatment markedly increased the transcripts of pro-inflammatory cytokine/chemokine (Il1b, Il1a, Il6, Ccl2, Ccl5) and inflammatory signaling mediators Tlr2 and Myd88 in the BM MSC (Fig. 7d). RNA-seq analysis also revealed significantly increased expression of collagen (Col3a1) in IL-1β -treated MSC (Fig. 7c). RT-qPCR and immunofluorescence staining also showed that IL-1α or IL-1β treatment significantly increased Col3a1 expression and addition of anti-IL-1R1 Ab significantly inhibited IL-1α or IL-1β induced Col3a1 expression in the BM MSC (Fig. 7e–g). Overall, these data suggest that blockade of IL-1R1 using anti-IL-1R1 antibody could diminish collagen deposition in the bone marrow.

To further understand the signaling downstream of IL-1/IL-1R1, we performed signaling studies using BM MSCs. We observed increased phosphorylation of p38 MAPK, p65 NF-κB, and JNK (c-Jun N-terminal Kinase) in MSCs treated with IL-1α or IL-1β (Supplementary Fig. 10a). We also compared the effects of IL-1β and TGF-β on cell signaling in BM MSCs. We observed more pronounced phosphorylation of p38 MAPK and p65 NF-κB by IL-1β stimulation compared with TGFβ (Supplementary Fig. 10b). Phosphorylation of Smad2 was induced by TGF-β but not by IL-1β stimulation, while JNK phosphorylation was induced by both IL-1β and TGF-β stimulation in MSCs (Supplementary Fig. 10b).

### Blocking of IL-1 receptor using anti-IL-1R1 antibody ameliorates BM fibrosis in a Jak2V617F mouse model of MF

**Fig. 8:**
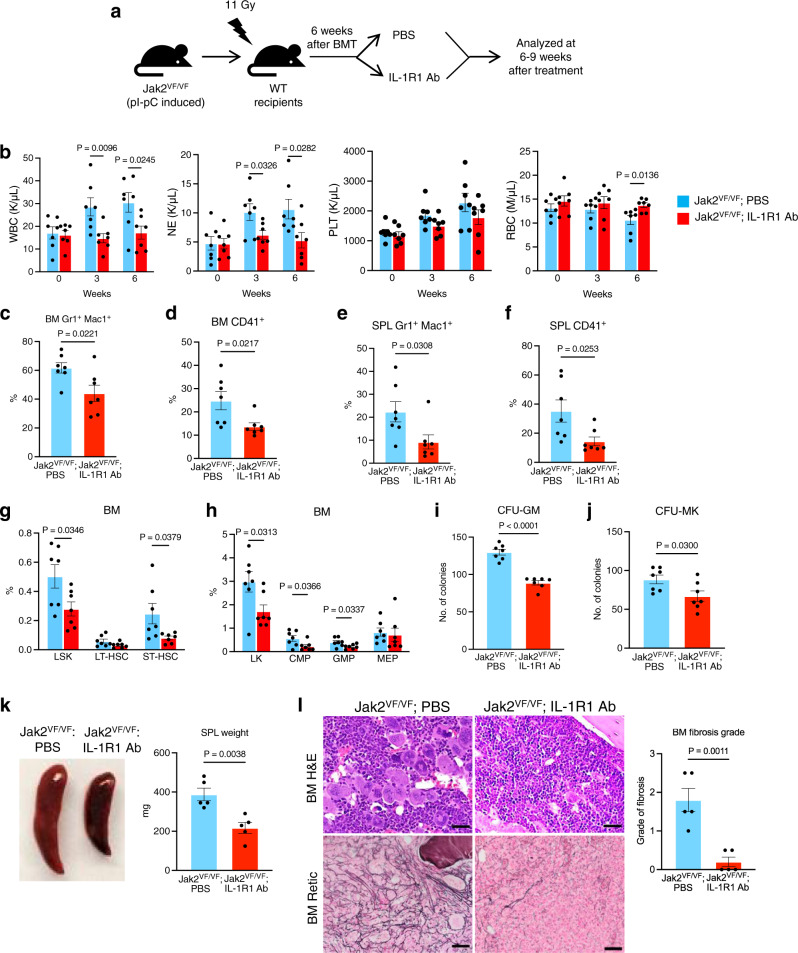
Treatment with anti-IL-1R1 antibody ameliorates BM fibrosis in homozygous Jak2V617F mouse model. **a** A scheme on the experimental design is depicted. **b** Peripheral blood WBC, neutrophil (NE), platelet (PLT) and red blood cell (RBC) counts were assessed at 3 and 6 weeks after the treatment (*n* = 7 mice per group). **c**, **d** Frequencies of Gr1^+^Mac1^+^ and CD41^+^ cells in the BM of Jak2^VF/F^ mice treated with PBS and IL-1R1 Ab (*n* = 7 mice per group). **e**, **f** Frequencies of Gr1^+^Mac1^+^ and CD41^+^ cells in the spleens (SPL) of Jak2^VF/VF^ mice treated with PBS and IL-1R1 Ab (*n* = 7 mice per group). **g** Frequencies of LSK, LT-HSC and ST-HSC in the BM of Jak2^VF/VF^ mice treated with PBS and IL-1R1 Ab (*n* = 7 mice per group). Results are shown in bar graphs as mean ± SEM. **h** Frequencies of LK, CMP, GMP and MEP in the BM of Jak2^VF/F^ mice treated with PBS and IL-1R1 Ab (*n* = 7 mice per group). **i**, **j** CFU-GM (**i**) and CFU-Mk (**j**) colonies in the BM of Jak2^VF/VF^ mice treated with PBS and IL-1R1 Ab (*n* = 7 mice per group). **k** Spleen size/weight in Jak2^VF/VF^ mice treated with vehicle (PBS) and IL-1R1 Ab are shown (*n* = 5 mice per group). **l** Representative images of the hematoxylin and eosin (H&E) and reticulin stained BM sections from Jak2^VF/F^ mice treated with PBS or IL-1R1 Ab. Scale bars, 20 μm. Histological grade of BM fibrosis is shown in bar graphs as mean ± SEM (*n* = 5 mice per group). Statistical significances were determined using multiple unpaired two-tailed *t*-tests. Source data are provided as a Source Data file.

Next, we tested the effects of blocking IL-1R1 using anti-IL-1R1 antibody in the homozygous Jak2V617F knock-in mouse model of MF. BM cells from homozygous Jak2V617F knock-in mice (Mx1Cre; Jak2^VF/VF^) at 6 weeks after pI-pC induction were transplanted into lethally irradiated C57BL/6 mice to generate a cohort of mice expressing Jak2^VF/VF^. At six weeks after BMT, mice were randomized into two groups to treat with vehicle (PBS) or anti-IL-1R1 antibody (IL-1R1 Ab) (3 μg/dose) by intraperitoneal injection 3 times a week for 6–9 weeks (as outlined in Fig. 8a). As expected, vehicle (PBS)-treated mice exhibited increased WBC, neutrophil and platelet counts in their peripheral blood (Fig. 8b). IL-1R1 Ab treatment significantly reduced WBC and neutrophil counts in these animals (Fig. 8b). IL-1R1 Ab treatment also reduced platelet counts although it did not reach statistical significance (Fig. 8b). RBC counts were reduced overtime in PBS treated Jak2^VF/VF^ mice while treatment with IL-1R1 Ab improved RBC counts in these animals (Fig. 8b). Flow cytometric analysis showed significant reduction in Gr-1^+^ Mac1^+^ and CD41^+^ cells in the BM and spleens of Jak2^VF/VF^ mice treated with IL-1R1 Ab compared with vehicle treatment (Fig. 8c–f). We also observed a significant reduction in LSK, ST-HSC, LK (Lin^-^c-Kit^+^) as well as common myeloid progenitors (CMP) and granulocyte-macrophage progenitors (GMP) in the BM of Jak2^VF/VF^ mice treated with IL-1R1 Ab compared with vehicle treatment (Fig. 8g, h). Hematopoietic progenitor colony assays showed a significant reduction of CFU-GM and CFU-Mk colonies in the BM of Jak2^VF/VF^ mice treated with IL-1R1 Ab (Fig. 8i, j). IL-1R1 Ab treatment also significantly reduced splenomegaly in Jak2^VF/VF^ mice (Fig. 8k). H&E staining showed increased clusters of abnormal megakaryocytes in the BM of vehicle-treated Jak2^VF/VF^ mice; however, they were significantly reduced by IL-1R1 Ab treatment (Fig. 8l). Reticulin staining showed extensive fibrosis in vehicle-treated Jak2^VF/VF^ mice BM (Fig. 8l). IL-1R1 Ab treatment significantly reduced BM fibrosis in Jak2^VF/VF^ mice (Fig. 8l). Collectively, these data suggest that blockade of IL-1R1 can inhibit BM fibrosis.

We also examined the effects of anti-IL-1R1 antibody treatment in C57BL/6 WT mice. We treated C57BL/6 WT mice with vehicle (PBS) or anti-IL-1R1 antibody (IL-1R1 Ab) (3 μg/dose) by intraperitoneal injection 3 times a week for 6 weeks (outlined in Supplementary Fig. 11a). We observed increases in WBC and neutrophil counts in IL-1R1 Ab treated WT animals compared to PBS treated animals although the values are still in the normal range for wild type animals (Supplementary Fig. 11b). However, we did not notice any significant differences in peripheral blood RBC, hemoglobin and platelet counts between PBS and IL-1R1 Ab treated groups (Supplementary Fig. 11b). Flow cytometric analyses also did not show any significant changes in Gr-1^+^ Mac1^+^, CD71^+^/Ter119^+^ and CD41^+^ cells in the BM and spleens of IL-1R1 Ab treated WT mice compared to PBS treated WT mice (Supplementary Fig. 11c–e). We also did not observe significant changes in LSK, LT-HSC, ST-HSC, LK (Lin^-^c-Kit^+^) as well as CMP, GMP and MEP in the BM and spleens of WT mice treated with IL-1R1 Ab compared to vehicle (PBS) treatment (Supplementary Fig. 11f, g). Hematopoietic progenitor colony assays showed no significant differences in BM-derived CFU-GM and BFU-E colonies between PBS and IL-1R1 Ab treated animals (Supplementary Fig. 11h, i). Spleen weights were comparable between PBS and L-1R1 Ab treated WT animals (Supplementary Fig. 11j). H&E staining of the BM sections also did not reveal any significant changes in BM histology between PBS and IL-1R1 Ab treated WT mice (Supplementary Fig. 11k).

## Discussion

In this study, we investigated the role of IL-1 in MPN pathogenesis since IL-1 is a key regulator of inflammation^10,11^ and the expression of IL-1 is significantly elevated in MPNs, in particular MF^7,15,16^. Consistent with previous reports^7,15,16^, we found elevated levels of IL-1α and IL-1β in patients with MF as well as in Jak2V617F knock-in mice. To determine the contribution of IL-1 signaling in the pathogenesis of MPN/MF, we examined the effects of genetic deletion of IL-1R1 in a homozygous Jak2V617F (Jak2^VF/VF^) knock-in mouse model of myelofibrosis. We demonstrate that deletion of IL-1R1 significantly reduced WBC, neutrophil and platelet counts and ameliorated BM fibrosis in homozygous Jak2V617F (Jak2^VF/VF^) knock-in mouse model of myelofibrosis. However, deletion of IL-1R1 in wild type background did not display any significant changes in blood counts and bone marrow progenitors, consistent with previous reports indicating that mice with IL-1α, IL-1β or IL-1R1 deletion do not exhibit defects in normal hematopoietic development^12,13,26^. We also found that deletion of IL-1R1 preferentially inhibited the expansion of Jak2V617F mutant HSPC and their progenies. Moreover, exogenous administration of IL-1β into heterozygous Jak2V617F knock-in mice (Jak2^VF/+^) resulted in the expansion of myeloid cells and progression to BM fibrosis. Taken together, these results suggest a pathogenic role of IL-1 signaling in clonal expansion of Jak2V617F mutant HSPC and progression to BM fibrosis.

We also investigated the contribution of BM microenvironmental IL-1 signaling in Jak2V617F-induced MPN. Prx1Cre-mediated deletion of IL-1R1 in the BM mesenchymal stromal cells (MSC) attenuated Jak2V617F-induced BM fibrosis without significantly altering hematopoiesis. Similar to this observation, a recent study found that disruption of TGF-β signaling in the BM MSC attenuates MPLW515L-induced BM fibrosis without affecting hematopoietic phenotypes in MPN^36^. We also showed that Prx1Cre-mediated deletion of IL-1R1 in BM MSCs significantly reduced collagen Col3a1 expression induced by IL-1α and IL-1β. It has been suggested that Gli1+ MSCs are a key driver of BM fibrosis^37^. We observed that both WT and Prx1Cre; IL-1R1F/F mice BM MSCs express Gli1, implying that Gli1+ MSCs can be targeted by the Prx1Cre. It has been reported that IL-1β induces damage to the BM microenvironmental cells that allows expansion of JAK2 mutant HSPC^38^. Our data indicate that selective disruption of IL-1 signaling in the BM microenvironment attenuates Jak2V617F-induced BM fibrosis without affecting hematopoietic phenotypes, whereas hematopoietic disruption of IL-1 signaling reduces both MPN hematopoietic phenotypes and BM fibrosis.

Transcriptome analysis of IL-1β-treated Jak2^VF/+^ mice LSK cells revealed enrichment of genes related to myeloid cell development, consistent with the notion that IL-1 signaling promotes the expansion of myeloid lineage cells in MPN. We observed significantly increased mRNA expression of Lcn2, Clec5a, Odc1, Bcat1 and Eif4a1 in IL-1β-treated Jak2^VF/+^ mice LSK cells (Fig. 6e). Interestingly, expression of these target genes is also significantly upregulated in MF patient’s CD34 + cells (Supplementary Fig. 9b). Lcn2 (Lipocalin 2) has been found elevated in patients with MF, and is suggested to promote proliferation of MF hematopoietic progenitors^39^. Clec5a is associated with myeloid differentiation^40^. Odc1 is a MYC target that is overexpressed in various cancers^41^. Bcat1 expression is upregulated in blast phase CML and AML and increased Bcat1 contributes to myeloid leukemia progression^42^. Eif4a1 is an eukaryotic translation initiation factor that promotes cell growth and cancer progression^43^. We found that ectopic expression of Lcn2, Clec5a or Bcat1 in the BM of Jak2^VF/+^ mice significantly increased myeloid (CFU-GM) colonies and enhanced megakaryocytic proliferation ex vivo (Fig. 6f, g). Thus, it is plausible that IL-1 induced increased expression of these target genes may contribute to enhanced cell proliferation and myeloid cell expansion in MPN.

We also observed increased expression of cytokines/chemokines and inflammatory signaling mediators by IL-1β treatment in MSCs. It has been reported that IL-1α and IL-1β can induce the expression of their own genes (Il1a and Il1b), which serves as positive feedback loop^44^. IL-1 also has been shown to induce other cytokines/chemokines such as, Il6 and Ccl2^44^. Myd88 is an important mediator of IL-1/IL-1R signaling^44^. Tlr2 and Myd88 are also components of innate immune signaling and have been implicated in tissue fibrosis^45,46^. Thus, IL-1 signaling in BM microenvironment may further amplify the inflammatory response via increased expression of cytokines/chemokines and signaling mediators that can promote remodeling of BM microenvironment. Our results also establish a direct link between IL-1 signaling and collagen expression in BM MSC. We observed that IL-1 treatment increases Col3a1 expression. Furthermore, blocking of IL-1R1 using anti-IL-1R1 antibody almost completely inhibited IL-1α or IL-1β induced Col3a1 expression in BM MSC. IL-1 can induce phosphorylation of p38 MAPK, p65 NF-κB and JNK^44^. A previous study suggested an association of NF-κB activation in MPN^47^. Activation of p38 MAPK was also observed in MF hematopoietic cells^48^. A recent report has indicated a role for JNK activation in myelofibrosis induced by MPLW515L^36^. We observed increased phosphorylation of p38 MAPK, p65 NF-κB and JNK in BM MSCs upon stimulation with IL-1α or IL-1β. Thus, it is plausible that activation of p38 MAPK, p65 NF-κB and JNK signaling downstream of IL-1 may contribute to remodeling of BM microenvironment and progression of MPN. Further studies are needed to delineate the contribution of p38 MAPK, p65 NF-κB and JNK activation downstream of IL-1 signaling in Jak2V617F-driven myelofibrosis.

Data from our IL-1R1 deletion studies prompted us to investigate the effects of blocking IL-1R1 signaling in a Jak2V617F mouse model. Similar to the effects of IL-1R1 deletion, anti-IL-1R1 antibody treatment significantly decreased leukocytosis and splenomegaly and markedly reduced BM fibrosis in homozygous Jak2V617F mice. Unlike JAK inhibitor ruxolitinib, which does not eliminate Jak2 mutant HSPC or significantly reduce BM fibrosis^5^, anti-IL-1R1 antibody treatment significantly reduced Jak2V617F mutant HSPC and ameliorated BM fibrosis (Fig. 7). Consistent with this finding, a previous study suggested that inhibition of IL-1 signaling using IL-1R antagonist enhanced elimination of CML leukemic stem cells^49^.

In conclusion, we demonstrate that IL-1 signaling contributes to clonal expansion of Jak2V617F mutant HSPC and progression of bone marrow fibrosis in MPN. We also show that both hematopoietic and BM microenvironmental IL-1 signaling influence the progression to bone marrow fibrosis. Furthermore, we show that blocking of IL-1 signaling significantly improves BM fibrosis in a Jak2V617F mouse model of MF. Similar observations have been made in a study by Dr. Skoda and colleagues^50^. Results from our studies suggest that therapies targeting IL-1R1 could be useful for the treatment of myelofibrosis.

## Methods

### Mice

Conditional Jak2V617F knock-in^22^, Mx1Cre^25^, IL-1R1 floxed^26^, Prx1Cre^32^ and UBC-GFP^51^ mice were previously described. Mx1Cre expression was induced by intraperitoneal injection of polyinosine-polycytosine (pI-pC). All animal studies were approved by the Institutional Animal Care and Use Committee of the University of Virginia School of Medicine. All mice were bred and maintained under a pathogen-free, 12-h light/dark cycles environment at the University of Virginia animal facility. The housing temperature is between 20–21 °C and the humidity is between 40–60%. All experiments were conducted with age- and sex-matched mice in a C57BL/6 background.

### Patient samples

Peripheral blood samples from MPN patients were collected at the University of Virginia Cancer Center. Informed consent was obtained for sample collection according to the protocols approved by the institutional review board of the University of Virginia Health System and in accordance with the Declaration of Helsinki. Patients were not specifically recruited for this study. MPN patient blood samples were obtained from the University of Virginia Biorepository.

### Cytokine analysis

IL-1α and IL-1β levels in the serum of mice were determined using ELISA kits (R&D Systems) according to the manufacturer’s protocols. IL-1α and IL-1β levels in the plasma of healthy controls and patients with PV and MF were determined by Luminex.

### Bone marrow transplantation

For BM transplantation (BMT) assay, BM cells (1 x 10^6^) from 6 weeks old WT, Mx1Cre; IL-1R1^F/F^, Mx1Cre; Jak2^VF/VF^ and Mx1Cre; Jak2^VF/VF^; IL-1R1^F/F^ mice were transplanted into lethally irradiated C57BL/6 recipient mice. For competitive repopulation assays, BM cells from uninduced Mx1Cre; Jak2^VF/+^ and Mx1Cre; Jak2^VF/+^; IL-1R1^F/F^ mice (CD45.2^+^) were mixed with CD45.1^+^ competitor BM cells at a ratio of 1:1 and then transplanted into lethally irradiated (CD45.1^+^) congenic mice. Four weeks after transplantation, recipients were injected with 5 doses of pI-pC. The chimerism in the BM of transplanted animals was assessed by CD45.2 and CD45.1 expression using flow cytometry. To study the contribution of IL-1 signaling in BM microenvironment on Jak2V617F driven MPN, BM cells (1 × 10^6^) from Jak2^VF/VF^ mice were transplanted into irradiated (10 Gy) WT or Prx1Cre; IL-1R1^F/F^ recipient mice. For competitive repopulation assay in Prx1Cre; IL-1R1^F/F^ recipient mice, BM cells from Jak2^VF/+^; GFP mice were mixed with WT BM cells at a ratio of 1:1 and then transplanted into irradiated (10 Gy) WT or Prx1Cre; IL-1R1^F/F^ recipient mice. The chimerism in the BM of transplanted animals was assessed by GFP expression. For IL-1R1 Ab treatment, BM from homozygous Jak2V617F (Jak2^VF/VF^) were transplanted into lethally-irradiated C57BL/6 recipient mice. At 6 weeks after transplantation, mice expressing Jak2^VF/VF^ were given 3 μg of IL-1R1 Ab (#AF771, R&D Systems) intraperitonially 3 times a week for 6–9 weeks.

### Plasmids

Lcn2 (RDC2923) and Celec5a (RDC2597) cDNA ORF constructs were purchased from R&D systems and sub-cloned into pCDH vector. Bcat1 (MG52932-UT) cDNA ORF clone was purchased from Sino Biological US Inc and sub-cloned into MSCV-IRES-GFP vector.

### Bone marrow transduction and Mk cell proliferation

Bone marrow cells were transduced with lentiviruses expressing vector, Lcn2, Celec5a, or retroviruses expressing Bcat1. Infected cells were selected using puromycin (2.5 μg/mL) for 48 h. Selected cells were cultured in StemPro-34 SFM (Thermo fisher) medium supplemented with nutrients, 2mM L-glutamate, Tpo (50 ng/mL) and SCF (20 ng/mL) for 4 days to establish megakaryocytic (Mk) culture. Equal number of Mk cells were plated and cell proliferation was assessed every 2 days for 6 days using viable cell counts or CellTiter-Glo (Promega).

### Colony-forming assays

BM (2 × 10^4^) cells were plated in duplicate in cytokine-supplemented complete methylcellulose medium (MethoCult M3434; Stem Cell Technologies). Burst forming units-erythroid (BFU-E) colonies and granulocyte-macrophage colony-forming units (CFU-GM) colonies were scored on day 7. To detect colony-forming units-megakaryocyte (CFU-Mk), BM cells were plated in collagen-based MegaCult medium (Stem Cell Technologies) in the presence of IL-3, IL-6, IL-11, and Tpo. CFU-Mk colonies were scored after 7–8 days according to manufacturer’s protocol. In some cases, BM cells from WT, Jak2V617F and IL-1R1cKO mice were plated in the presence of IL-1β or IL-1α and CFU-GM and CFU-Mk colonies were assessed.

### Flow cytometry

Single-cell suspensions were prepared from BM and spleen, and red blood cells were lysed with red blood cell lysis solution. Cells were washed and resuspended in PBS plus 2% FBS and stained for 30 min on ice with directly conjugated monoclonal antibody specific for Ter119, CD71, CD41, CD61, Mac-1, Gr-1, CD45R (B220), or TCRβ. For HSC/progenitor analysis, BM cells were stained for 60 min on ice with antibody against c-Kit, Sca-1, Flk2 (CD135), CD34, CD16/32 (FcγR II/III), and antibody against lineage (Lin) markers including CD3e, CD4, CD8, CD19, B220, Gr-1, Ter119, and IL-7R (CD127). To distinguish between donor-derived and recipient hematopoietic cells in competitive bone marrow transplantation experiments, PE-CD45.1 and FITC-CD45.2 conjugated antibodies were used for flow cytometry. Antibody information, supplier name, catalog number and dilution information are available in the Supplementary Table 3. Flow cytometry was performed with the Cytek Aurora or Cytek Northern Lights (Cytek Biosciences) and SpectroFlo v3.0.3 software (Cytek Biosciences). Flow cytometry data were analyzed using FlowJo 10 software (FlowJo, LLC). Gating strategy for HSC/progenitor analysis are included in Supplementary Fig. 3.

### Real-time quantitative PCR

Total RNA was extracted from mice BM MSCs and sorted LSK (Lin^−^Sca-1^+^c-kit^+^) and LK (Lin^-^c-kit^+^) cells using RNeasy Mini or Micro kit (Qiagen) and cDNA samples were prepared by using QuantiTect Reverse Transcription kit (Qiagen). Real-time quantitative PCR was performed on Quantstudio3 system (Applied Biosystems) using SYBR Green PCR master mix (Quantabio). The data were normalized to *Hprt1* and fold changes in mRNA expression were determined by the ΔΔCt method. Sequences of the primers are available in the Supplementary Table 2.

### Immunoblotting

Mouse BM MSC were starved in serum free medium for 6 h followed by IL-1α, IL-1β or TGF-β stimulation for 30 min. Cells were lysed with RIPA lysis buffer containing protease inhibitors. Immunoblotting was performed using phospho-specific antibody against p-p38 (#9211, Cell Signaling), p-p65 (#3033, Cell Signaling), p-JNK (#9251, Cell Signaling), p-Smad2 (#3108, Cell Signaling), and total p38 (#9212, Cell Signaling), p65 (#sc-372, Santa Cruz Biotechnology), JNK (sc-474, Santa Cruz Biotechnology), Smad2 (#5339, Cell Signaling). β-Actin (#A5441, Sigma) was used as loading control. Antibody information, supplier name, catalog number and dilution information are available in the Supplementary Table 3.

### MSC culture and immunofluorescence staining

Mesenchymal stromal cells (MSCs) were generated from WT and Prx1Cre; Il1-1R1^F/F^ (IL-1R1 KO) mice as previously described^52^. For immunofluorescence staining, MSCs were grown on cover slips. MSCs were incubated with IL-1α/IL-1β (5 ng/ml) or IL-1α/IL-1β + IL-1R1 Ab (1 μg/ml) for 72 h. Collagen staining was performed with un-conjugated antibody against Col3a1 (#ab7778 Abcam). Secondary staining was done using TRITC goat anti-rabbit antibody (#111-025-003, Jackson Immunoresearch). Fluorescence was visualized using Zeiss LAM 710 Confocal microscope. Data were analyzed using Image J software.

### RNA-sequencing analysis

LSK and LK cells were sorted from Jak2^VF/+^ mice treated with PBS or IL-1β at 16 weeks after treatment using a FACS Aria II. Total RNA was extracted from LSK and LK cells using RNeasy micro kit (Qiagen). BM MSCs were treated with PBS or IL-1β for 72 h and total RNA was extracted using RNeasy Mini kit (Qiagen). RNA sequencing was performed using NextSeq 500 High Output Kit and NextSeq 500 sequencing instrument (Illumina). RNA-seq data alignment was performed using UCSC mm10 reference genome with HISAT2 V2.0.1. The read counts and differential analysis were done using Genomic Alignments and DESeq2. P-adjusted value of <0.05 with a log_2_fc > 0.5 was considered as significant change in gene expression.

### Blood and tissue analysis

Peripheral blood counts were determined using Hemavet 950FS (Drew Scientific). For histopathologic analysis, mouse tissue specimens were fixed in 10% neutral buffered formalin and embedded in paraffin. Tissue sections (4 μm) were stained with hematoxylin and eosin (H&E) and reticulin stain. Histopathology analyses and fibrosis grading were performed by a specialized hematopathologist blinded to group assignment. Bone marrow fibrosis was graded from 0 to 3. In order to be precise, fibrosis gradings 0, 0.5, 1.0, 1.5, 2.0, 2.5 and 3.0 were used.

### Statistical analysis

Statistical analysis was performed using the GraphPad Prism 9.4.1 (GraphPad Software). One-way or two-way ANOVA with Tukey’s multiple comparison test was used when comparing more than two groups. For comparisons between two groups, unpaired two-tailed Student’s t-test was used. All data are presented as mean ± SEM. *P* < 0.05 was considered statistically significant. All experiments were repeated with sufficient reproducibility.

### Reporting summary

Further information on research design is available in the Nature Research Reporting Summary linked to this article.

## Supplementary information

Supplementary Information

Reporting Summary

## Data Availability

The RNA-sequencing data generated in this study have been deposited to the NCBI GEO database under the accession number GSE180339. Source data are provided as a Source Data file. Publicly available gene expression datasets (GSE166629, GSE53482, EGAD00001004788) were also used for analysis. The remaining data are available within the Article, Supplementary Information or Source Data file. Source data are provided with this paper.

## Source data

Source Data
