# Supplementary material for: Interleukin-1 contributes to clonal expansion and progression of bone marrow fibrosis in JAK2V617F-induced myeloproliferative neoplasm
Source: Nat Commun. 2022 Sep 13;13:5347. doi: 10.1038/s41467-022-32928-3 (PMC9470702; doi:10.1038/s41467-022-32928-3)
Supplement: Supplementary file 1 — Supplementary Information [file 41467_2022_32928_MOESM1_ESM.pdf]

Supplementary Information

Interleukin-1 contributes to clonal expansion and progression of bone marrow fibrosis in JAK2V617F-induced myeloproliferative neoplasm

Rahman et al.,

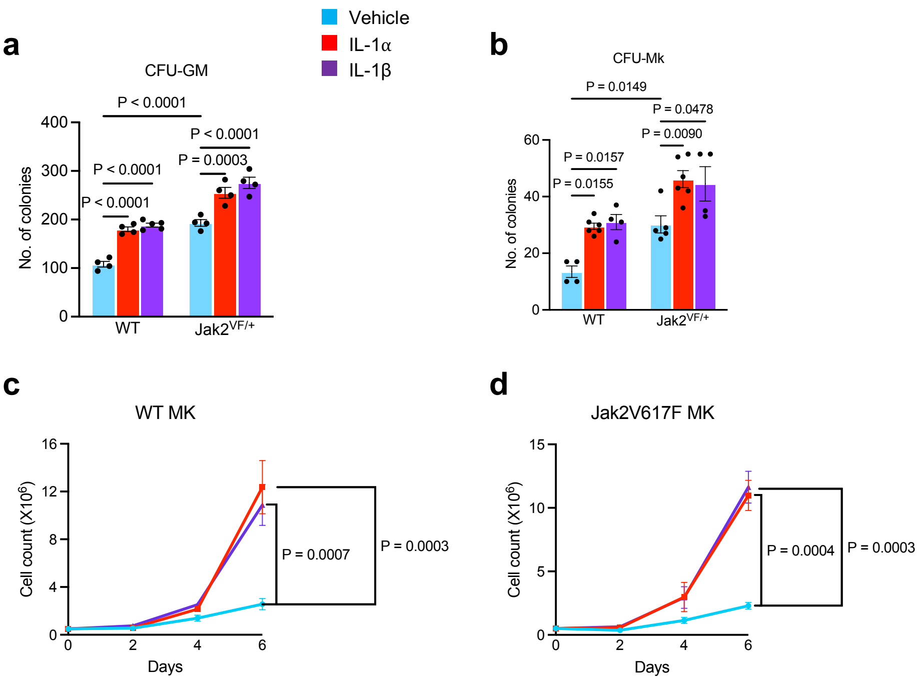

Supplementary Fig. 1. IL-1 stimulation enhances myeloid and megakaryocytic colony formation and cell proliferation. a Bone marrow (BM) cells from WT and Jak2V617F mice were plated in complete methylcellulose medium in the presence of vehicle (PBS), IL-1α (10 ng/mL) or IL-1β (10 ng/mL). Colony-forming unit granulocyte-macrophage (CFU-GM) colonies were assessed. Data are shown in bar graphs as mean± SEM (n=4, 4, 5 biological replicates for vehicle, IL-1α and IL-1β treatment in WT mice BM; n=4, 4, 4 biological replicates for vehicle, IL-1α and IL-1β treatment in $Jak2^{VF/+}$ mice BM). **b** BM cells from WT and Jak2V617F mice were plated in complete MegaCult medium in the presence of vehicle, IL-1α (10 ng/mL) or IL-1β (10 ng/mL). Megakaryocytic colonies (CFU-Mk) were assessed. Data are shown in bar graphs as mean± SEM (n=4, 6, 4 biological replicates for vehicle, IL-1α and IL-1β treatment in WT mice BM; n=5, 6, 4 biological replicates for vehicle, IL-1α and IL-1β treatment in $Jak2^{VF/+}$ mice BM). Statistical significance was determined using two-way ANOVA with Tukey's multiple comparison test. **c,** Megakaryocytic (Mk) cell proliferation. Megakaryocytes derived from WT BM were cultured in the presence of vehicle, IL-1α (10 ng/mL) or IL-1β (10 ng/mL). Mk cell proliferation was assessed by viable cell counts every 2 days for 6 days. (n=9, 9, 9, 8 biological replicates for vehicle, n=8, 8, 6, 8 for IL-1α and n=9, 9, 6, 8 for IL-1β treatment at 0, 2, 4 and 6 days). **d** Megakaryocytic (Mk) cell proliferation in $Jak2^{VF/+}$ mice BM in the presence of vehicle, IL-1α (10 ng/mL) or IL-1β (10 ng/mL) (n=4 biological replicates for each condition at different time points). Statistical significance was determined using multiple unpaired two-tailed t-tests. Source data are provided as a Source Data file.

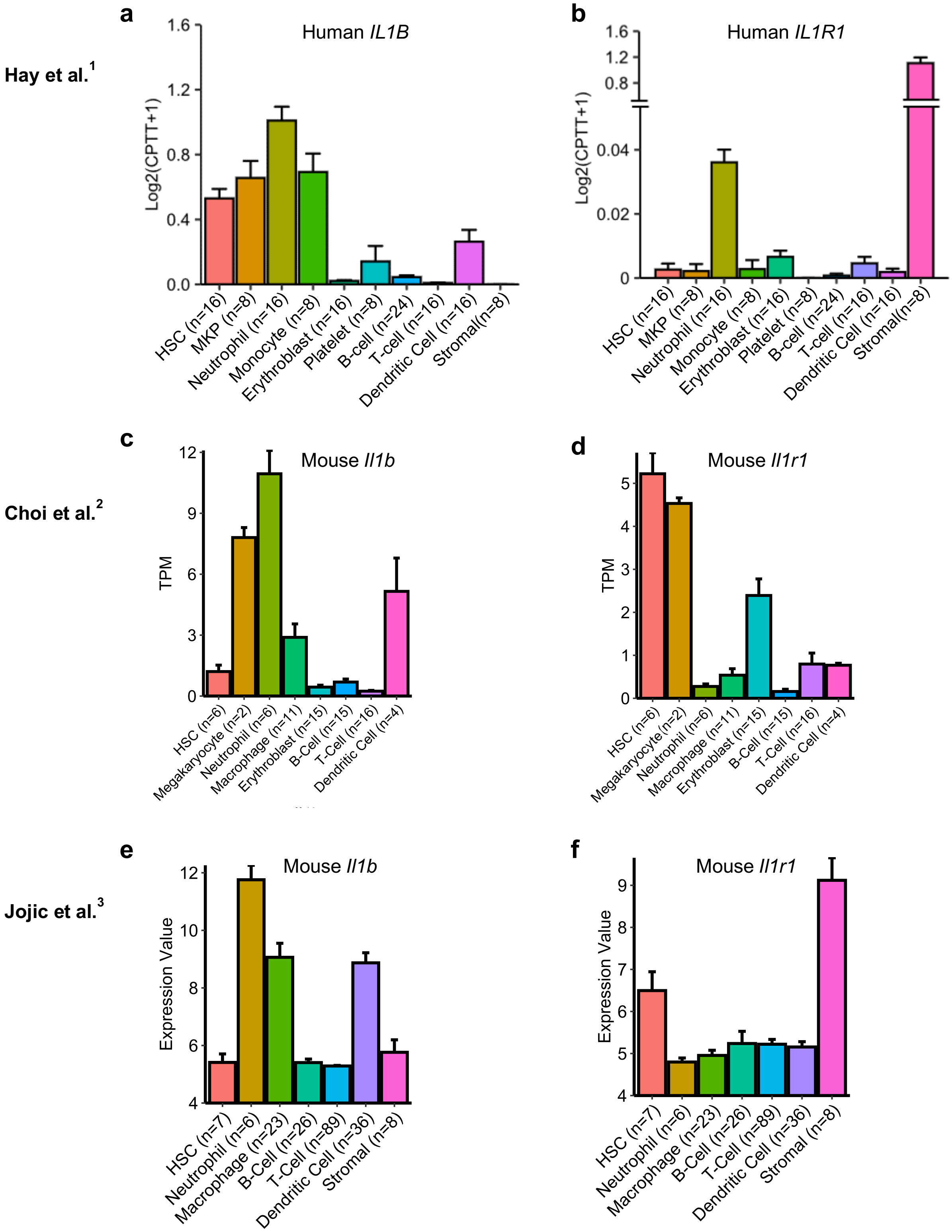

Supplementary Fig. 2. Expression levels of IL-1β and IL-1R1 in human and mouse hematopoietic compartments and stromal cells. a, b Analysis of publicly available human bone marrow single-cell RNA-sequencing data[1] showed the expression of *IL1B* and *IL1R1* in various hematopoietic cells and stromal cells. *IL1B* and *IL1R1* expression values are shown as $\log_2$ count per ten thousand (CPTT) +1.

c, d Analysis of publicly available RNA-sequencing data on mouse bone marrow[2] showed the expression levels *Il1b* and *Il1r1* mRNA in different hematopoietic cells. The *Il1b* and *Il1r1* expression values are shown as Transcripts Per Million (TPM). **e, f** Analysis of microarray gene-expression data on mouse bone marrow[3] showed the expression of *Il1b* and *Il1r1* in various hematopoietic cells and stromal cells. The number (n) of biological samples for each cell type is shown in the figure panels.

Gating strategy for HSC/progenitors:

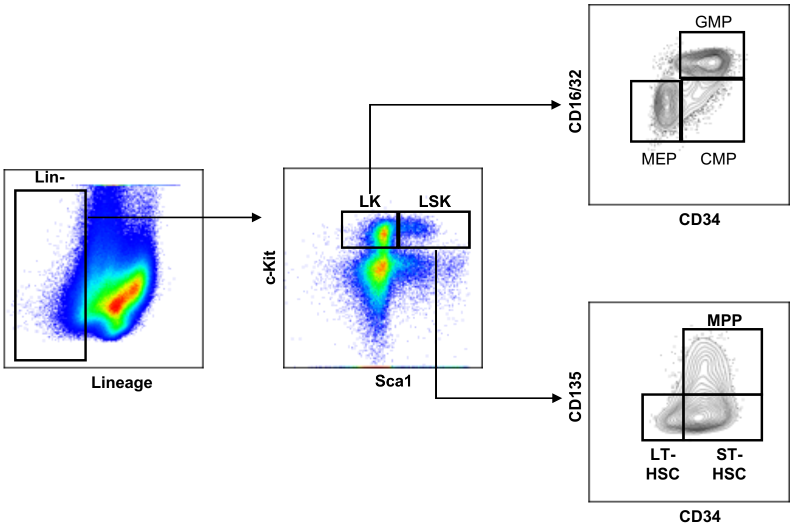

Supplementary Fig. 3. FACS gating strategies for mouse hematopoietic stem/progenitor cells. Lineage negative (Lin^-) cells were first gated from the total bone marrow and spleen cells. Lin^- cells were then gated into LSK (Lin^- $Sca1^+$ $c\text{-}kit^+$) and LK ($Lin^-c\text{-}kit^+$). LSK cells were subclassified into LT-HSC (Lin^- $Sca1^+$ $c\text{-}kit^+$ $CD34^-$ $CD135^-$), ST-HSC (Lin^- $Sca1^+$ $c\text{-}kit^+$ $CD34^+$ $CD135^-$) and MPP (Lin^- $Sca1^+$ $c\text{-}kit^+$ $CD34^+$ $CD135^+$). LK cells were subclassified into CMP ($Lin^-c\text{-}kit^+CD34^+CD16/32^{lo}$), GMP ($Lin^-c\text{-}kit^+CD34^+CD16/32^{hi}$) and MEP ($Lin^-c\text{-}kit^+CD34^-CD16/32^-$).

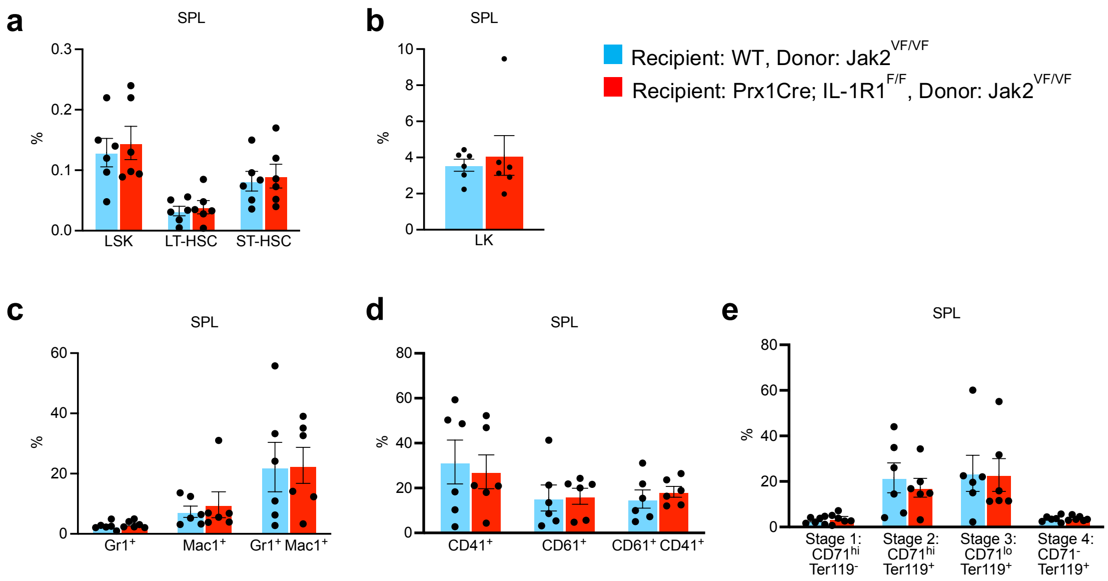

Supplementary Fig. 4. Flow cytometric analysis of hematopoietic compartments in Prx1Cre; IL-1R1$^{F/F}$ recipient mice in a non-competitive setting. a, b Frequencies of LSK, LT-HSC, ST-HSC and LK cells in the spleens (SPL) of WT and Prx1Cre; IL-1R1$^{F/F}$ recipient mice are shown in bar graphs as mean± SEM (n= 6 mice per group). **c-e** Frequencies of Gr1^{+}/Mac1^{+} **(c)**, CD61^{+}/CD41^{+} **(d)** and CD71/Ter119 **(e)** cells in the spleens from WT and Prx1Cre; IL-1R1$^{F/F}$ recipients are shown in bar graphs as mean± SEM (n= 6 mice per group). Multiple unpaired two-tailed t-tests were performed. No significant difference was observed between the two groups. Source data are provided as a Source Data file.

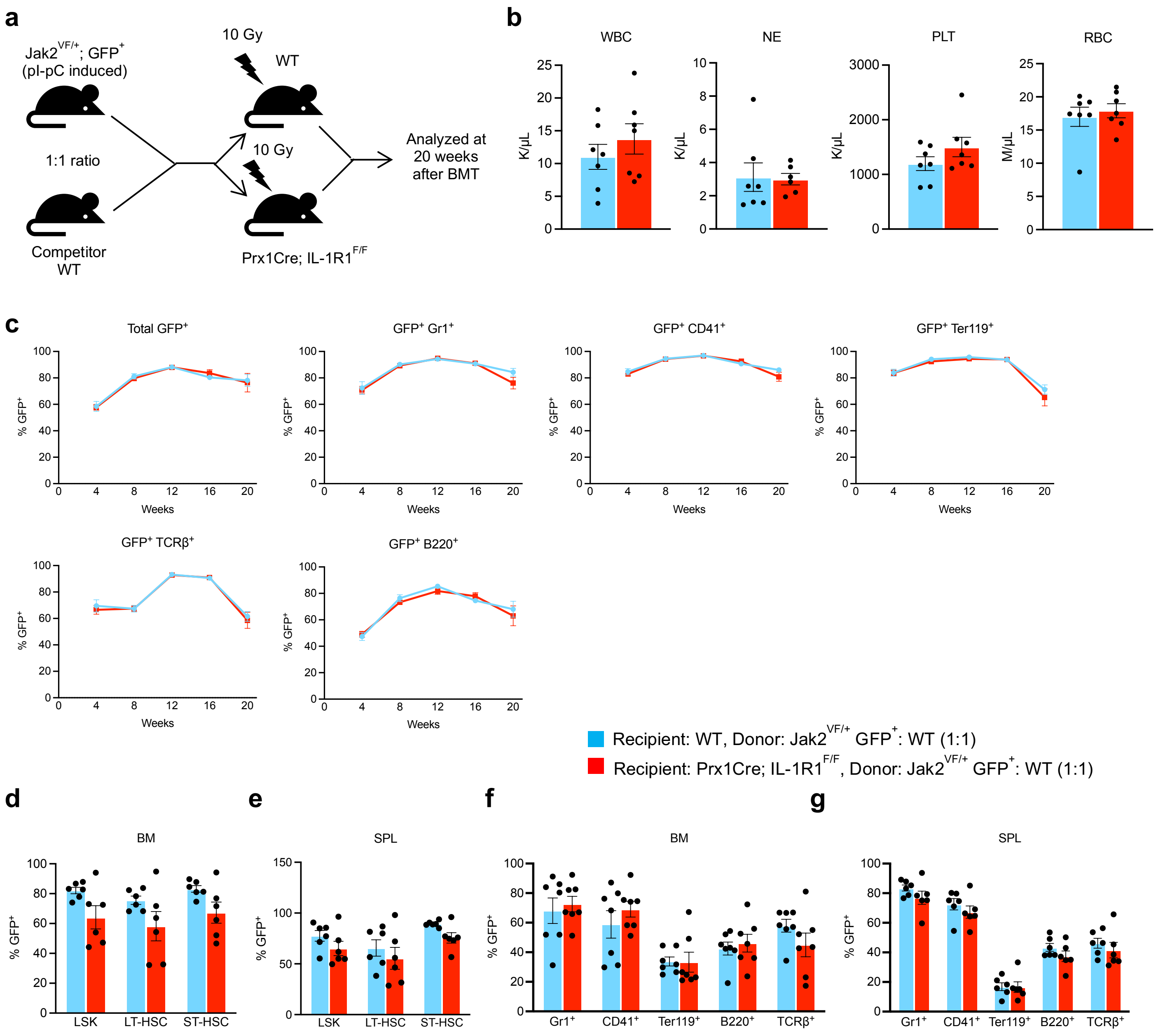

Supplementary Fig. 5. Flow cytometric analysis of hematopoietic compartments in Prx1Cre; IL-1R1$^{F/F}$ recipient mice in a competitive setting. **a** Schematic design of competitive BM transplantation into WT and Prx1Cre; IL-1R1$^{F/F}$ recipient mice is shown. **b** Peripheral blood WBC (n=7, 7), NE (n=7, 6), PLT (n=7, 7) and RBC (n=7, 7) counts were assessed at 20 weeks after transplantation. **c** Percentages of Jak2V617F mutant (GFP$^+$) total cells, GFP$^+$ Gr1$^+$, GFP$^+$ CD41$^+$, GFP$^+$ Ter119$^+$, GFP$^+$ B220$^+$ and GFP$^+$ TCRβ$^+$ cells in the peripheral blood of WT and Prx1Cre; IL-1R1$^{F/F}$ recipients were assessed (n=9, 9, 8, 8, 6 for WT recipient mice, n=9, 9, 9, 8, 6 for Prx1Cre; IL-1R1$^{F/F}$ recipient mice at 4, 8, 12, 16 and 20 weeks after transplantation). **d, e** Percentages of Jak2V617F mutant (GFP$^+$) LSK, LT-HSC and ST-HSC in the BM **(d)** and spleens **(e)** of recipient mice at 20 weeks after transplantation are shown as mean ± SEM (n=6 mice per group). **f, g** Percentages of Jak2V617F mutant (GFP$^+$) Gr1$^+$, CD41$^+$, Ter119$^+$, B220$^+$ and TCRβ$^+$ cells in the BM (n= 7 mice per group) **(f)** and spleens (SPL) (n= 6 mice per group) **(g)** from

WT and Prx1Cre; IL-1R1$^{F/F}$ recipient animals are shown as mean ± SEM. Statistical comparisons were performed using multiple unpaired two-tailed t-tests. No significant difference was observed between the two groups. Source data are provided as a Source Data file.

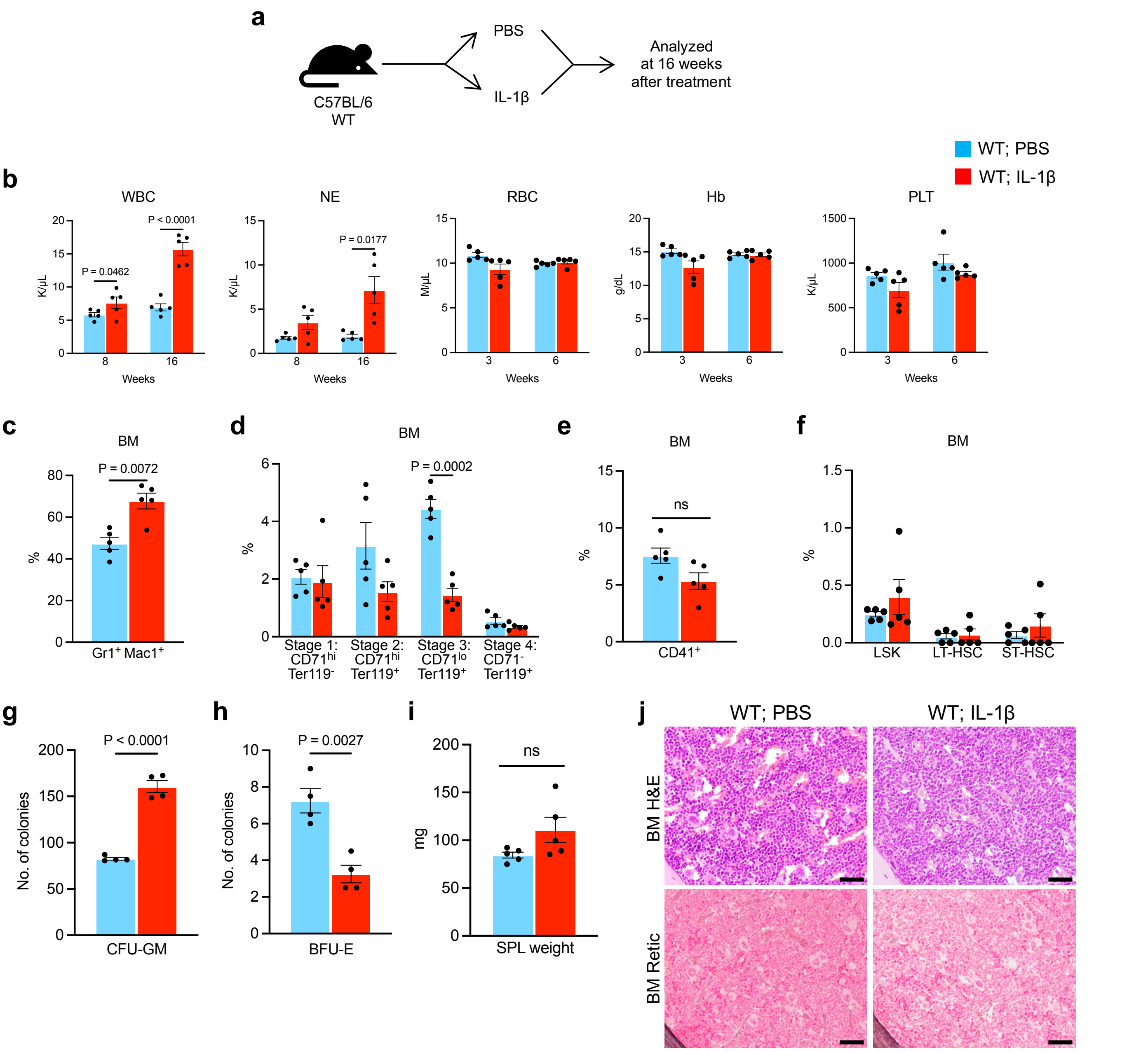

Supplementary Fig. 6. Effects of exogenous IL-1β treatment on WT mice. a Schematic design of the experimental approach is depicted. C57BL/6 WT mice were treated with either PBS or IL-1β (0.5 μg/dose; 3 doses/week) via intraperitoneal (IP) injection for 16 weeks. **b** Peripheral blood WBC, neutrophil (NE), red blood cells (RBC), hemoglobin (Hb) and platelets (PLT) were assessed at 8 and 16 weeks after treatment (n=5 mice per group). **c-e** Frequencies of $Gr1^+/Mac1^+$ **(c)**, $CD71^+/Ter119^+$) **(d)** and $CD41^+$ **(e)** cells in the BM of C57BL/6 mice treated with PBS or IL-1β are shown in bar graphs as mean ± SEM (n=5 mice per group). **f** Frequencies of LSK, LT-HSC and ST-HSC in the BM of C57BL/6 mice treated with PBS or IL-1β are shown in bar graphs as mean ± SEM (n=5 mice per group). **g, h** CFU-GM **(g)** and BFU E **(h)** colonies in the BM of WT mice treated with PBS and IL-1β are shown in bar graphs as mean ± SEM (n=4 mice per group; each data point is an average of two technical replicates). **i** Spleen weights in PBS and IL-1β treated C57BL/6 mice (n=5 mice per group). **j** Representative images of hematoxylin and eosin

(H&E) and reticulin stained BM sections from C57BL/6 WT mice treated with PBS or IL-1β are shown (n=5 mice per group). Scale bar, 20 μm. Statistical significance was determined using multiple unpaired two-tailed t-tests. Source data are provided as a Source Data file.

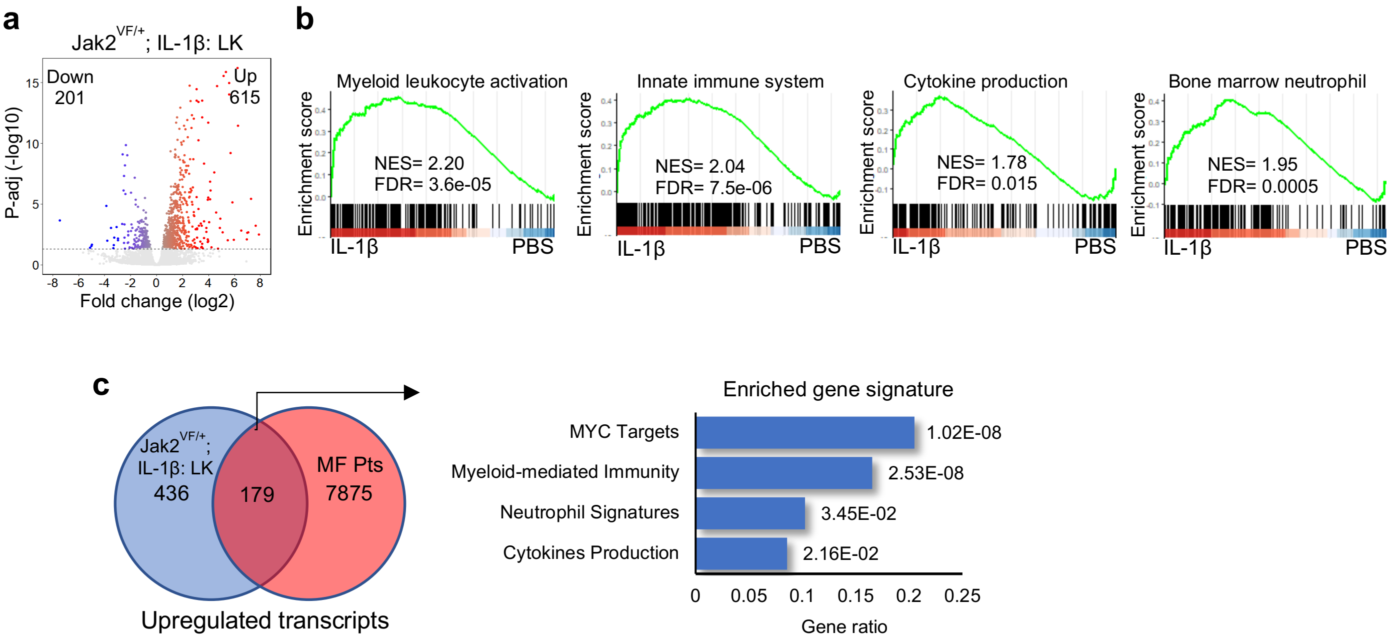

Supplementary Fig. 7. Effects of IL-1β on gene expression in Jak2V617F mice myeloid progenitors. a Volcano plot showing significantly upregulated and downregulated (p-adj < 0.05 and $log_2fc > 0.5$) genes in LK ($Lin^-c\text{-}kit^+$) cells isolated from $Jak2^{VF/+}$ mice treated with vehicle (PBS) (n=3) or IL-1β (n=2). **b** GSEA show significant alterations of genes related to myeloid leukocyte activation, innate immune system, cytokine production and bone marrow neutrophils in IL-1β treated $Jak2^{VF/+}$ LK cells (n=2) compared to PBS treated $Jak2^{VF/+}$ LK cells (n=3). Enrichment plots with normalized enrichment score (NES) and false discovery rate (FDR) are shown. **c** Venn diagram showing the overlap between upregulated genes in MF patient granulocytes (n=62 for MF, n=23 for control) and genes upregulated in IL-1β treated $Jak2^{VF/+}$ LK cells (n=2 for IL-1β treated, n=3 for PBS treated). The cutoffs were FDR-adjusted p < 0.05. Overlapping genes showed enrichment for MYC targets, myeloid-leukocyte mediated immunity, cytokine production and bone marrow neutrophil gene signatures.

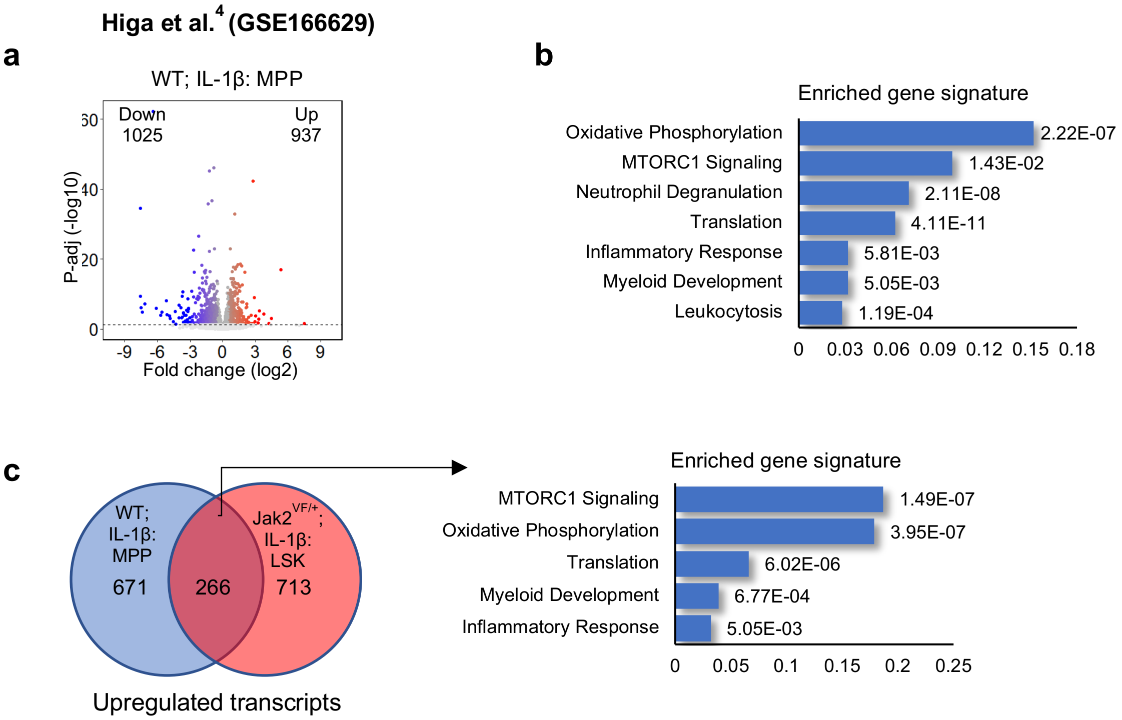

Supplementary Fig. 8. Effects of IL-1β on gene expression in WT mice hematopoietic progenitors. **a** Analysis of publicly available RNA-seq dataset (GSE166629)[4] from IL-1β treated WT mouse multipotent progenitor (MPP) cells. Volcano plot showing significantly upregulated and downregulated (p-adj < 0.05 and $\log_2$fc >0.5) genes in IL-1β treated WT mice MPP cells (n=6) compared to PBS treated WT MPP cells (n=6). **b** Gene-set enrichment analyses (GSEA) show significant alterations of genes related to oxidative phosphorylation, mTORC1 signaling, neutrophil degranulation, translation, inflammatory response, myeloid development and leukocytosis in IL-1β treated WT MPP cells compared with PBS treated WT MPP cells. Enrichment gene signatures/pathways with normalized enrichment score (NES) and false discovery rate (FDR) are shown. **c** Venn diagram showing the overlap between upregulated genes in Jak2$^{VF/+}$ LSK cells (n=2 for IL-1β treated, n=3 for PBS treated) and genes upregulated in IL-1β treated WT MPP cells (n=6 for IL-1β treated, n=6 for PBS treated). The cutoffs were FDR-adjusted p < 0.05. Overlapping genes showed enrichment for mTORC1 signaling, oxidative phosphorylation, translation, myeloid development and inflammatory response gene signatures.

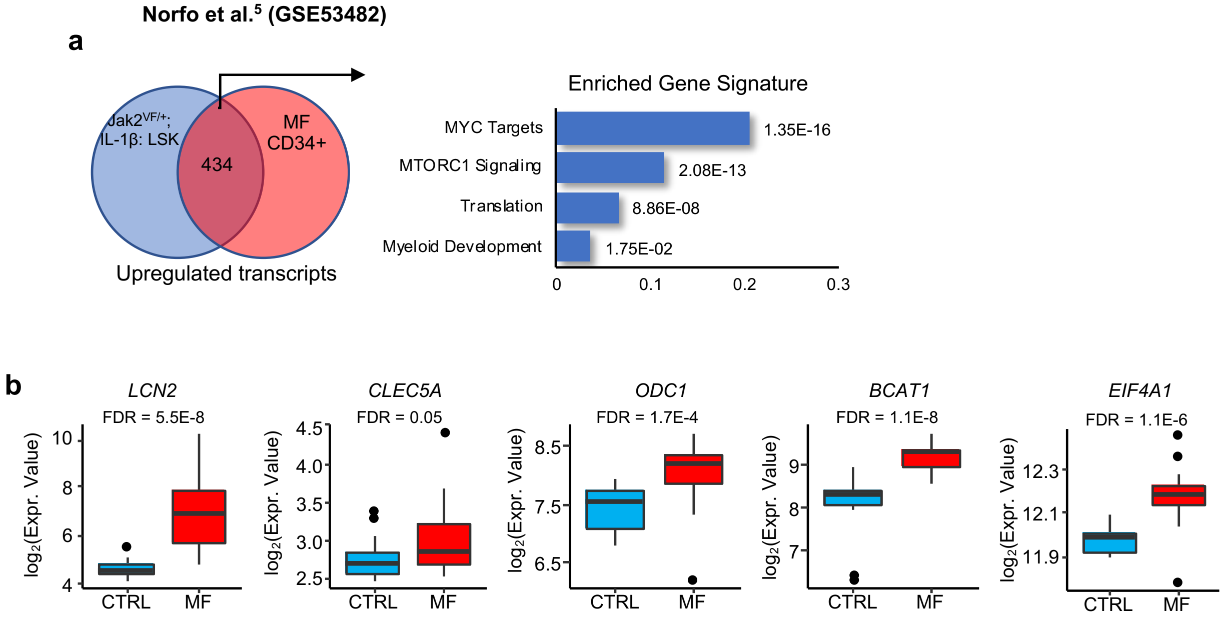

Supplementary Fig. 9. Comparison of the gene expression between IL-1β treated Jak2$^{VF/+}$ LSK and MF CD34+ cells. a Venn diagram showing the overlap between upregulated genes in IL-1β treated Jak2$^{VF/+}$ LSK cells and the genes upregulated in MF patient CD34+ cells[5]. The cutoffs were FDR-adjusted $p < 0.05$. Overlapping genes showed enrichment for MYC targets, mTORC1 signaling, translation and myeloid development gene signatures. **b** Expression of *LCN2*, *CLEC5A*, *ODC1*, *BCAT1* and *EIF4A1* mRNA was significantly increased in MF CD34+ cells compared to heathy controls (n=16 for controls, n=42 for MF). Expression values are shown as $\log_2$ of normalized count. Box plots display 1st and 3rd quartiles of the interquartile range (IQR) corresponding to 25th and 75th percentile and median center line. Whisker's maxima and minima are within 1.5*IQR. FDR-adjusted p values are shown.

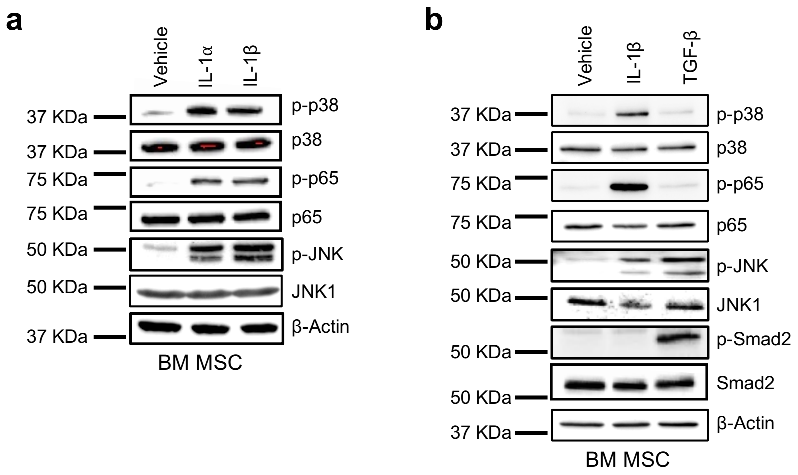

Supplementary Fig. 10. Effects of IL-1 stimulation on cell signaling in bone marrow mesenchymal stromal cells. a Immunoblotting was performed with indicated phospho-specific or total antibodies on cell lysates from mesenchymal stromal cells (MSCs) following stimulation with IL-1α (10 ng/mL) or IL-1β (10 ng/mL) for 30 min. Note that IL-1α or IL-1β stimulation increased phosphorylation of p38 MAPK (p-p38), p65 NF-κB (p-p65) and p-JNK in MSCs. β-Actin was used as a loading control. Blots shown are representative of three independent experiments. **b** Immunoblotting was performed with indicated phospho-specific or total antibodies on cell lysates from MSCs following stimulation with IL-1β (10 ng/mL) or TGF-β (10 ng/mL) for 30 min. Blots shown are representative of two independent experiments. Uncropped scans of the immunoblots are provided in the Supplementary Information.

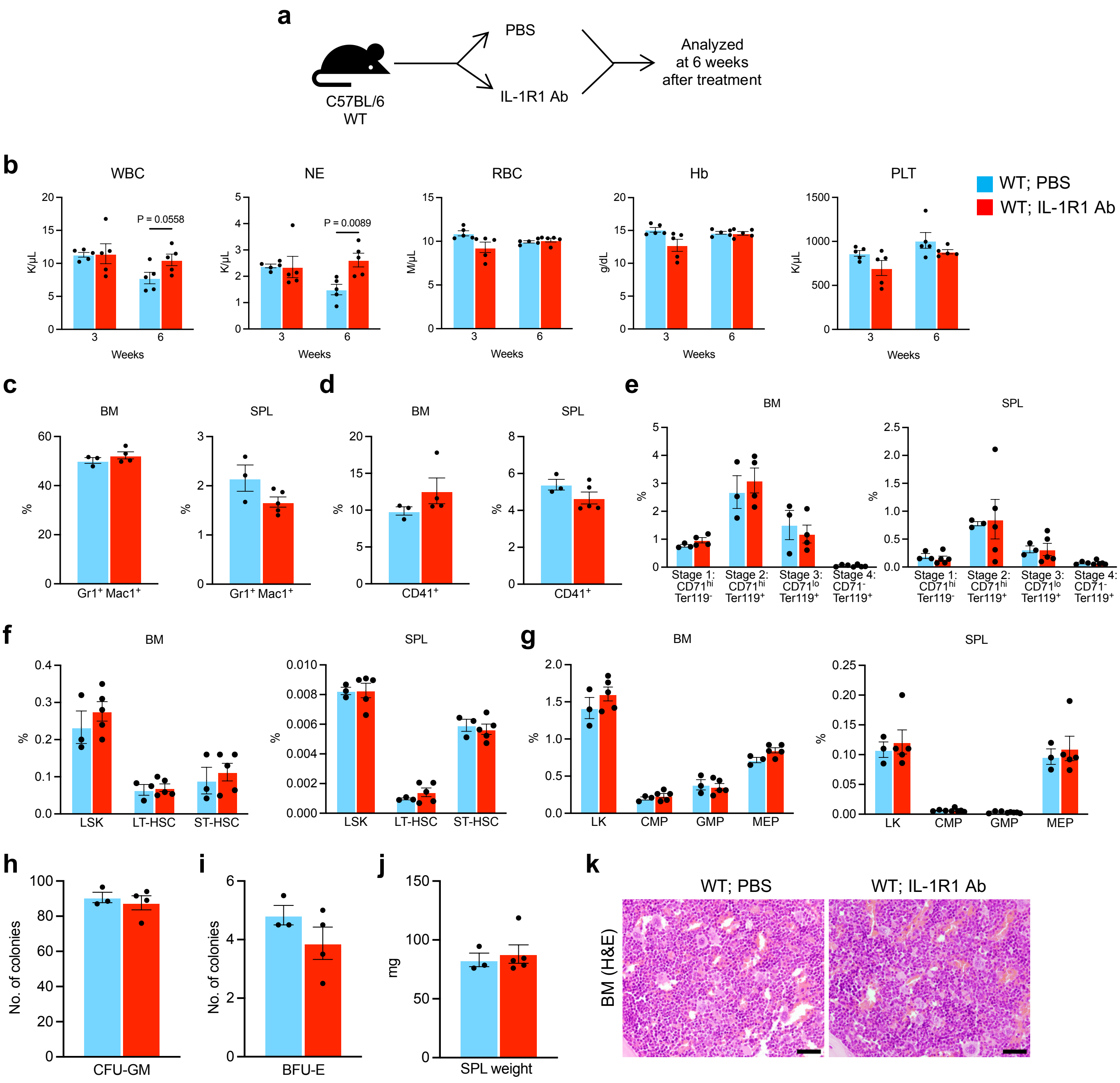

Supplementary Fig. 11. Effects of anti-IL-1R1 antibody treatment in C57BL/6 WT mice. a Schematic design of the experimental approach is depicted. C57BL/6 WT mice were treated with either vehicle (PBS) or anti-IL-1R1 antibody (IL-1R1 Ab) at 3μg/dose/mouse, 3 doses/week for 6 weeks. **b** Peripheral blood WBC, neutrophil (NE), red blood cell (RBC), hemoglobin (Hb) and platelet (PLT) counts were assessed at 3 and 6 weeks after the treatment (n=5 mice per group). **c-e** Frequencies of Gr1$^+$/Mac1$^+$ **(c)**, CD41$^+$ **(d)**, and CD71/Ter119 **(e)** cells in the BM and spleens of WT mice treated with PBS or IL-1R1 Ab are shown in bar graphs as mean± SEM (n=3, 4 mice for BM; n= 3, 5 mice for spleen). **f** Frequencies of LSK, LT-HSC and ST-HSC in the BM and spleens of WT mice treated with PBS or IL-1R1 Ab (n=3, 5

mice). **g** Frequencies of LK, CMP, GMP and MEP in the BM and spleens of WT mice treated with PBS or IL-1R1 Ab (n=3, 5 mice). **h, i** CFU-GM **(h)** and BFU-E **(i)** colonies in the BM of WT mice treated with PBS and IL-1R1 Ab are shown in bar graphs as mean ± SEM (n=3, 4 mice, each data point is an average of two technical replicates). **j** Spleen weights in WT mice treated with vehicle (PBS) and IL-1R1 Ab are shown (n=3, 5 mice). **k** Representative images of the H&E stained BM sections from WT mice treated with vehicle (PBS) or IL-1R1 Ab are shown (n=3, 4 mice). Scale bars, 20 μm. Statistical significance was determined using multiple unpaired two-tailed t-tests. Source data are provided as a Source Data file.

Supplementary Table 1. A list of selected upregulated genes in MF patient granulocytes (p-adjusted values derived from the DESeq2 Wald Test are shown)

Symbol	Gene signature	log2FC	padj
LCN2	Myeloid Development	2.500	4.51E-06
CLEC5A	Myeloid Development	1.913	2.85E-09
TGFBI	Myeloid Development	2.384	1.85E-08
MPO	Myeloid Development	3.330	1.07E-06
ELANE	Myeloid Development	2.818	1.42E-05
CEBPA	Myeloid Development	0.372	0.15031872
ODC1	Myc Targets	0.515	0.03011034
POLD2	Myc Targets	1.045	0.00370835
CDC20	Myc Targets	2.407	1.61E-06
NPM1	Myc Targets	0.813	0.00992977
CCT2	Myc Targets	0.907	0.00026713
NHP2	Myc Targets	0.948	0.00659819
SRM	Myc Targets	1.045	0.0172233
SNRPD1	Myc Targets	0.605	0.01117277
BCAT1	mTORC1 Signaling	1.871	0.0001397
LDHA	mTORC1 Signaling	0.527	0.01098606
ASNS	mTORC1 Signaling	1.038	0.00059902
XBP1	mTORC1 Signaling	0.527	0.0278073
RRP9	mTORC1 Signaling	1.081	0.00655933
ETF1	mTORC1 Signaling	0.523	0.00174924
EIF4A1	Translation	0.914	2.74E-07
EIF2B3	Translation	1.006	0.02038894
YARS1	Translation	0.591	0.00053068
TARS1	Translation	0.630	0.00798021
MRPL19	Translation	0.775	0.01829895

Supplementary Table 2. Primers used for real-time quantitative PCR

Gene	Sequence (5' to 3')
Bcat1_Foward	TAGAATGTGCCGATCTGCTG
Bcat1_Reverse	CTTTGGAAGGCTTCTTGACG
Ccl2_Foward	CAGCCAGATGCAGTTAACGC
Ccl2_Reverse	TTCTTGGGGTCAGCACAGAC
Ccl5_Foward	GCTGCTTTGCCTACCTCTCC
Ccl5_Reverse	TCGAGTGACAAACACGACTGC
Clec5a_Foward	CCACTAGTGTGCAGAATGTCTC
Clec5a_Reverse	CCAAGGTGATTCGGAGAAGGA
Col3a1_Foward	GACCAAAAGGTGATGCTGGACAG
Col3a1_Reverse	CAAGACCTCGTGCTCCAGTTAG
Eif4a1_Foward	GGAAGGCGTCATCGAGAGTA
Eif4a1_Reverse	AATAGCTCGCTGCTGGATGG
Hprt1_Foward	CAACGGGGGACATAAAAGTTATTGGTGGA
Hprt1_Reverse	TGCAACCTTAACCATTTTGGGGCTGT
Il-1α_Foward	AAGCAACGGGAAGATTCTGA
Il-1α_Reverse	ACTTCTGCCTGACGAGCTTC
Il-1β_Foward	GCCCATCCTCTGTGACTCAT
Il-1β_Reverse	AGGCCACAGGTATTTTGTCG
Il6_Foward	TCCAGTTGCCTTCTTGGGAC
Il6_Reverse	GACAGGTCTGTTGGGAGTGG
lcn2_Foward	GGACTACAACCAGTTCGCCA
lcn2_Reverse	CTTGAGGCCCAGAGACTTGG
Myd88_Forward	AGAGCTGCTGGCCTTGTTAG
Myd88_Reverse	CGAAAAGTTCCGGCGTTTGT
Odc1_Foward	GATGGCCTTGATCGGATCGT
Odc1_Foward	TCTGCTTCATGAGTTGCCACA
Tlr2_Foward	GAGGTGCGGACTGTTTCCTT
Tlr2_Reverse	AGATTTGACGCTTTGTCTGAGG

Supplementary Table 3. List of antibodies used

Number	Antibody	Dilution	Assay
1	Anti-mouse IL-1R1 neutralizing Ab (#AF771, R&D Systems)	1 mg/mL	Confocal microscopy
2	p-p38 (#9211, Cell Signaling)	1:1000	Western blot
3	p-p65 (#3033, Cell Signaling)	1:1000	Western blot
4	p-JNK (#9251, Cell Signaling)	1:1000	Western blot
5	p-Smad2 (#3108, Cell Signaling)	1:1000	Western blot
6	Total p38 (#9212, Cell Signaling)	1:1000	Western blot
7	Total p65 (#sc-372, Santa Cruz Biotechnology)	1:1000	Western blot
8	JNK (sc-474, Santa Cruz Biotechnology)	1:1000	Western blot
9	Smad2 (#5339, Cell Signaling)	1:1000	Western blot
10	β-Actin (#A5441, Sigma)	1:10000	Western blot
11	Rabbit anti-mouse/human Col3a1 (#ab7778 Abcam)	1:200	Confocal microscopy
12	Goat anti-rabbit antibody, TRITC (#111-025-003, Jackson Immunoresearch)	1:200	Confocal microscopy
13	Anti-mouse CD3e Monoclonal Antibody (145-2C11), PerCP-Cyanine5.5 (#45-0031-82 ebiosciences)	1:100	Flow cytometry
14	Anti-mouse CD19 Antibody, PerCP/Cyanine5.5 (# 152406 Biolegend)	1:100	Flow cytometry
15	Anti-mouse CD8a Monoclonal Antibody (53-6.7), PerCP-Cyanine5.5 (# 45-0081-82 ebiosciences)	1:100	Flow cytometry
16	Anti-mouse CD19 Antibody, PerCP/Cyanine5.5 (# 152406 Biolegend)	1:100	Flow cytometry
17	Anti-mouse CD45R (B220) Monoclonal Antibody (RA3-6B2), PerCP-Cyanine5.5(#45-0452-82 ebiosciences)	1:100	Flow cytometry
18	Anti-mouse Ly-6G/Ly-6C (Gr-1) Antibody, PerCP/Cy5.5 (#108428 Biolegend)	1:100	Flow cytometry
19	anti-mouse CD127 (IL-7Ra) Antibody, PerCP/Cy5.5 (#135022 Biolegend)	1:100	Flow cytometry
20	Anti-mouse TER-119/Erythroid Cells Antibody, PerCP/Cy5.5 (# 116228 Biolegend)	1:100	Flow cytometry
21	Anti-mouse CD117 (c-kit) Antibody, APC/Cy7 (# 105826 Biolegend)	1:100	Flow cytometry
22	Anti-mouse Ly-6A/E (Sca-1) Monoclonal Antibody (D7), PE-Cyanine7 (# 25-5981-82 ebiosciences)	1:100	Flow cytometry
23	Anti-mouse CD135 (Flt3) Monoclonal Antibody (A2F10), APC (#17-1351-82 ebiosciences)	1:100	Flow cytometry
24	Anti-mouse CD34 Monoclonal Antibody (RAM34), FITC (#11-0341-82 ebiosciences)	1:100	Flow cytometry
25	Anti-mouse CD16/CD32 Monoclonal Antibody (93), PE (#12-0161-82 ebiosciences)	1:100	Flow cytometry
26	Anti-mouse F4/80 Monoclonal Antibody (BM8), APC, # 17-4801-82 eBioscience	1:200	Flow cytometry
27	Anti-mouse CD16/CD32 Monoclonal Antibody (93), PE (#12-0161-82 ebiosciences)	1:200	Flow cytometry
28	Anti-mouse Ly-6G/Ly-6C (Gr-1) Antibody, PerCP/Cy5.5 (#108428 Biolegend)	1:200	Flow cytometry
29	Anti-mouse/human CD11b Antibody, Brilliant Violet 785 (#101243 Biolegend)	1:200	Flow cytometry
30	Anti-mouse TER119/Erythroid Cells Antibody, FITC (#116206 Biolegend)	1:200	Flow cytometry
31	Anti-mouse CD71 Antibody, PE/Cyanine7 (#113812 Biolegend)	1:200	Flow cytometry
32	Anti-mouse CD45R (B220) Monoclonal Antibody (RA3-6B2), Alex Fluro 700, (#56-0452-82 ebiosciences)	1:200	Flow cytometry
33	Anti-mouse TCRb chain Antibody, Brilliant Violet 605 (#109241 Biolegend)	1:200	Flow cytometry
34	Anti-mouse APC/Cyanine anti-mouse CD41 Antibody, APC/Cyanine (#133928 Biolegend)	1:200	Flow cytometry
35	Anti-mouse TER-119/Erythroid Cells Antibody (#116212 Biolegend)	1:200	Flow cytometry
36	Anti-mouse CD71 Antibody (#113808 Biolegend)	1:200	Flow cytometry
37	Anti-mouse Ly-6G/Ly-6C (Gr-1) Antibody (#108428 Biolegend)	1:200	Flow cytometry
38	Anti-mouse/human n CD11b Antibody (#101259 Biolegend)	1:200	Flow cytometry
39	Anti-mouse CD41a Monoclonal Antibody (eBioMWReg30 (MWReg30), FITC (#11-0411-82 ebiosciences)	1:200	Flow cytometry
40	Anti-mouse/rat CD61 CD61 Antibody (#104318 Biolegend)	1:200	Flow cytometry
41	Anti-mouse/human CD45R/B220 Antibody (#103223 Biolegend)	1:200	Flow cytometry
42	Anti-mouse CD90.2 (Thy-1.2) Antibody (#140318 Biolegend)	1:200	Flow cytometry

Supplementary References

1. Hay, S. B., Ferchen K., Chetal K, Grimes H. L., Salomonis N. The Human Cell Atlas bone marrow single-cell interactive web portal. *Exp Hematol.* **68**, 51-61 (2018).
2. Choi, J. et al. Haemopedia RNA-seq: a database of gene expression during haematopoiesis in mice and humans. *Nucleic Acids Res.* **47**, D780-D785 (2019).
3. Jojic, V. et al. Identification of transcriptional regulators in the mouse immune system. *Nat Immunol.* **14**, 633-643 (2013).
4. Higa, K. C. et al. Chronic interleukin-1 exposure triggers selection for Cebpa-knockout multipotent hematopoietic progenitors. *J Exp Med.* **218**, e20200560 (2021).
5. Norfo, R. et al. miRNA-mRNA integrative analysis in primary myelofibrosis CD34+ cells: role of miR-155/JARID2 axis in abnormal megakaryopoiesis. *Blood.* **124**, e21-32 (2014).

Supplementary Figure 10a

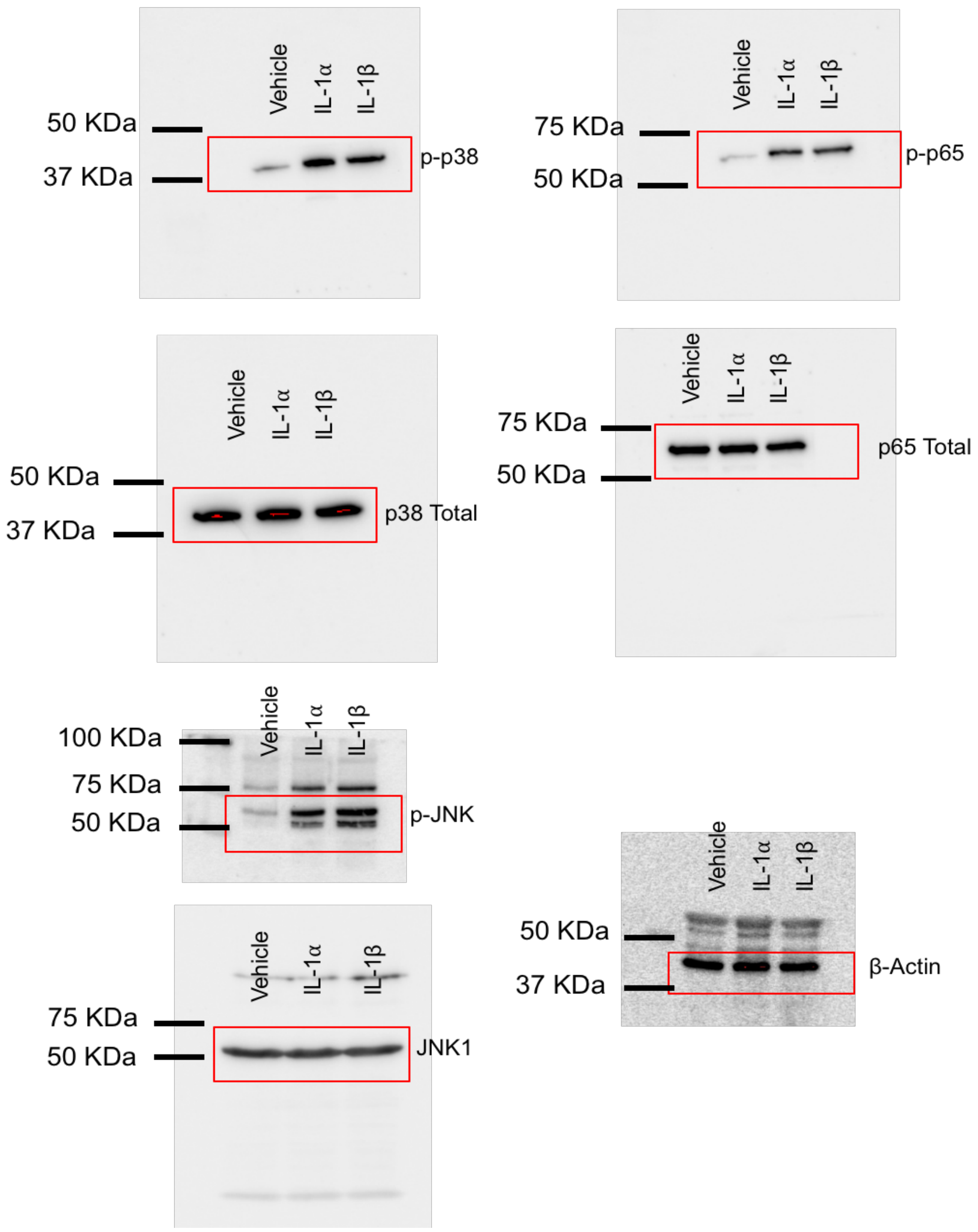

Supplementary Fig. 12. Uncropped scans of the immunoblots used in the Supplementary figure 10a.

Supplementary Figure 10b

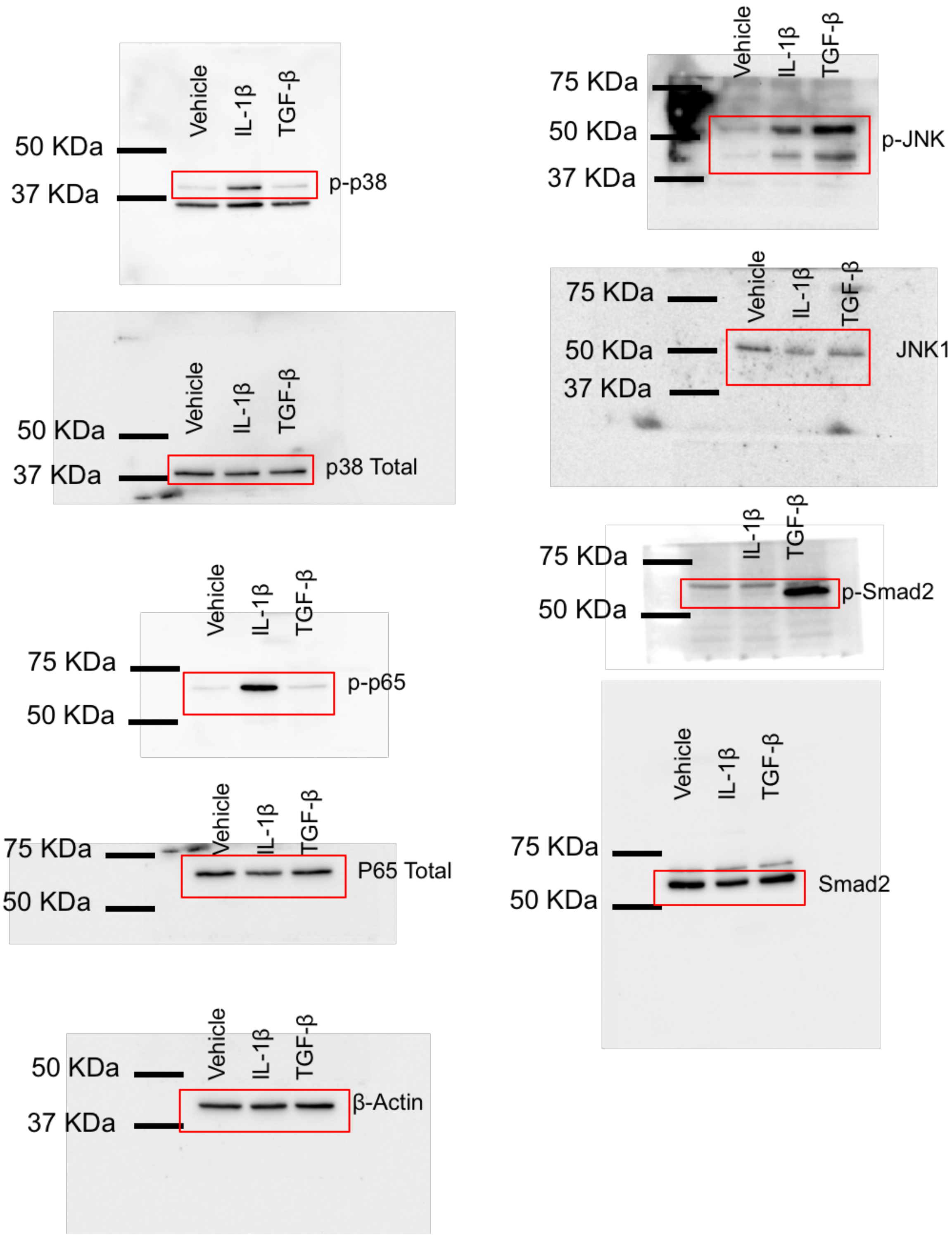

Supplementary Fig. 13. Uncropped scans of the immunoblots used in the Supplementary figure 10b.
