# Supplementary material for: Interleukin-1 contributes to clonal expansion and progression of bone marrow fibrosis in JAK2V617F-induced myeloproliferative neoplasm
Source: Nat Commun. 2022 Sep 13;13:5347. doi: 10.1038/s41467-022-32928-3 (PMC9470702; doi:10.1038/s41467-022-32928-3)
Supplement: Supplementary file 2 — Reporting Summary [file 41467_2022_32928_MOESM2_ESM.pdf]

nature portfolio

Corresponding author(s): Golam Mohi

Last updated by author(s): Aug 17, 2022

Reporting Summary

Nature Portfolio wishes to improve the reproducibility of the work that we publish. This form provides structure for consistency and transparency in reporting. For further information on Nature Portfolio policies, see our Editorial Policies and the Editorial Policy Checklist.

Statistics

For all statistical analyses, confirm that the following items are present in the figure legend, table legend, main text, or Methods section.

n/a	Confirmed	
☐	☒	The exact sample size (*n*) for each experimental group/condition, given as a discrete number and unit of measurement
☐	☒	A statement on whether measurements were taken from distinct samples or whether the same sample was measured repeatedly
☐	☒	The statistical test(s) used AND whether they are one- or two-sided<br>*Only common tests should be described solely by name; describe more complex techniques in the Methods section.*
☒	☐	A description of all covariates tested
☐	☒	A description of any assumptions or corrections, such as tests of normality and adjustment for multiple comparisons
☐	☒	A full description of the statistical parameters including central tendency (e.g. means) or other basic estimates (e.g. regression coefficient) AND variation (e.g. standard deviation) or associated estimates of uncertainty (e.g. confidence intervals)
☐	☒	For null hypothesis testing, the test statistic (e.g. *F*, *t*, *r*) with confidence intervals, effect sizes, degrees of freedom and *P* value noted<br>*Give P values as exact values whenever suitable.*
☒	☐	For Bayesian analysis, information on the choice of priors and Markov chain Monte Carlo settings
☒	☐	For hierarchical and complex designs, identification of the appropriate level for tests and full reporting of outcomes
☐	☒	Estimates of effect sizes (e.g. Cohen's *d*, Pearson's *r*), indicating how they were calculated

Our web collection on statistics for biologists contains articles on many of the points above.

Software and code

Policy information about availability of computer code

Data collection	SpectroFlo v3.0.3 software for Cytek Aurora or Cytek Northern Lights<br>QuantStudio3 Firmware v1.3.0 for qPCR<br>Luminex MAGPIX using Milliplex Analyst v5.1 software<br>Plate reader biotek synergy HT software v2.00.18<br>NovaSeq 6000 for RNA sequencing
Data analysis	Image J v1.53 for image analysis<br>GraphPad prism 9.4.1 and MS Excel for statistical analysis<br>FlowJo V10 for flow cytometry data analysis<br>For RNA sequencing data analysis, Unix programs: Fastp V0.20.0, HISAT2 V2.0.1, samtools V1.10, featureCounts V2.0.2<br>R packages: dplyr V1.0.6, data.table V1.14.0, DESeq2 V1.28.1, ensembldb V2.12.1, ggplot2 V3.3.5, clusterProfiler 3.16.1, enrichplot 1.8.1<br>Genome alignment: Mus Musculus GRCm38 build 100 were used.

For manuscripts utilizing custom algorithms or software that are central to the research but not yet described in published literature, software must be made available to editors and reviewers. We strongly encourage code deposition in a community repository (e.g. GitHub). See the Nature Portfolio guidelines for submitting code & software for further information.

Data

Policy information about availability of data

All manuscripts must include a data availability statement. This statement should provide the following information, where applicable:

- Accession codes, unique identifiers, or web links for publicly available datasets
- A description of any restrictions on data availability
- For clinical datasets or third party data, please ensure that the statement adheres to our policy

Source data are provided with this paper.
RNA sequencing data generated in this study have been deposited to the NCBI GEO database under the accession number GSE180339.
Publicly available gene expression datasets (GSE166629, GSE53482, EGAD00001004788) were also used for analysis.

Field-specific reporting

Please select the one below that is the best fit for your research. If you are not sure, read the appropriate sections before making your selection.

[x] Life sciences [ ] Behavioural & social sciences [ ] Ecological, evolutionary & environmental sciences

For a reference copy of the document with all sections, see nature.com/documents/nr-reporting-summary-flat.pdf

Life sciences study design

All studies must disclose on these points even when the disclosure is negative.

Sample size	No statistical method was used to predetermine the sample size. Sample sizes were chosen based on previous experience with similar type of experiments in our laboratory.
Data exclusions	No data were excluded.
Replication	All experiments were replicated independently. Number of repeats is provided in the figure legends.
Randomization	Animals were randomly divided into experimental and control groups. Cells were all plated at the same time and wells were randomly selected for different treatments.
Blinding	Investigators were not blinded to group allocation. Blinding was not possible as the same investigator performed mouse genotyping, treatment and end-point analysis.

Reporting for specific materials, systems and methods

We require information from authors about some types of materials, experimental systems and methods used in many studies. Here, indicate whether each material, system or method listed is relevant to your study. If you are not sure if a list item applies to your research, read the appropriate section before selecting a response.

Materials & experimental systems

n/a	Involved in the study	
[ ]	[x]	Antibodies
[x]	[ ]	Eukaryotic cell lines
[x]	[ ]	Palaeontology and archaeology
[ ]	[x]	Animals and other organisms
[ ]	[x]	Human research participants
[x]	[ ]	Clinical data
[x]	[ ]	Dual use research of concern

Methods

n/a	Involved in the study	
[x]	[ ]	ChIP-seq
[ ]	[x]	Flow cytometry
[x]	[ ]	MRI-based neuroimaging

Antibodies

Antibodies used	1) Anti-mouse IL-1R1 neutralizing Ab (#AF771, R&D Systems) [1 mg/ml]<br>2) p-p38 (#9211, Cell Signaling) [1:1000]<br>3) p-p65 (#3033, Cell Signaling) [1:1000]<br>4) p-JNK (#9251, Cell Signaling) [1:1000]<br>5) p-Smad2 (#3108, Cell Signaling) [1:1000]<br>6) Total p38 (#9212, Cell Signaling) [1:1000]<br>7) Total p65 (#sc-372, Santa Cruz Biotechnology) [1:1000]

8) JNK (sc-474, Santa Cruz Biotechnology) [1:1000]
9) Smad2 (#5339, Cell Signaling) [1:1000]
10) β-Actin (#A5441, Sigma) [1:10000]
11) Rabbit anti-mouse/human Col3a1 (#ab7778 Abcam) [1:200]
12) Goat anti-rabbit antibody, TRITC (#111-025-003, Jackson Immunoresearch) [1:200]
13) Anti-mouse CD3e Monoclonal Antibody (145-2C11), PerCP-Cyanine5.5 (#45-0031-82 ebiosciences) [1:100]
14) Anti-mouse CD19 Antibody, PerCP/Cyanine5.5 (# 152406 Biolegend) [1:100]
15) Anti-mouse CD8a Monoclonal Antibody (53-6.7), PerCP-Cyanine5.5 (# 45-0081-82 ebiosciences) [1:100]
16) Anti-mouse CD19 Antibody, PerCP/Cyanine5.5 (# 152406 Biolegend) [1:100]
17) Anti-mouse CD45R (B220) Monoclonal Antibody (RA3-6B2), PerCP-Cyanine5.5(#45-0452-82 ebiosciences) [1:100]
18) Anti-mouse Ly-6G/Ly-6C (Gr-1) Antibody, PerCP/Cy5.5 (#108428 Biolegend) [1:100]
19) anti-mouse CD127 (IL-7Ra) Antibody, PerCP/Cy5.5 (#135022 Biolegend) [1:100]
20) Anti-mouse TER-119/Erythroid Cells Antibody, PerCP/Cy5.5 (# 116228 Biolegend) [1:100]
21) Anti-mouse CD117 (c-kit) Antibody, APC/Cy7 (# 105826 Biolegend) [1:100]
22) Anti-mouse Ly-6A/E (Sca-1) Monoclonal Antibody (D7), PE-Cyanine7 (# 25-5981-82 ebiosciences) [1:100]
23) Anti-mouse CD135 (Flt3) Monoclonal Antibody (A2F10), APC (#17-1351-82 ebiosciences) [1:100]
24) Anti-mouse CD34 Monoclonal Antibody (RAM34), FITC (#11-0341-82 ebiosciences) [1:100]
25) Anti-mouse CD16/CD32 Monoclonal Antibody (93), PE (#12-0161-82 ebiosciences) [1:100]
26) Anti-mouse F4/80 Monoclonal Antibody (BM8), APC, # 17-4801-82 eBioscience [1:200]
27) Anti-mouse CD16/CD32 Monoclonal Antibody (93), PE (#12-0161-82 ebiosciences) [1:200]
28) Anti-mouse Ly-6G/Ly-6C (Gr-1) Antibody, PerCP/Cy5.5 (#108428 Biolegend) [1:200]
29) Anti-mouse/human CD11b Antibody, Brilliant Violet 785 (#101243 Biolegend) [1:200]
30) Anti-mouse TER119/Erythroid Cells Antibody, FITC (#116206 Biolegend) [1:200]
31) Anti-mouse CD71 Antibody, PE/Cyanine7 (#113812 Biolegend) [1:200]
32) Anti-mouse CD45R (B220) Monoclonal Antibody (RA3-6B2), Alex Fluro 700, (#56-0452-82 ebiosciences) [1:200]
33) Anti-mouse TCRb chain Antibody, Brilliant Violet 605 (#109241 Biolegend) [1:200]
34) Anti-mouse APC/Cyanine anti-mouse CD41 Antibody, APC/Cyanine (#133928 Biolegend) [1:200]
35) Anti-mouse TER-119/Erythroid Cells Antibody (#116212 Biolegend) [1:200]
36) Anti-mouse CD71 Antibody (#113808 Biolegend) [1:200]
37) Anti-mouse Ly-6G/Ly-6C (Gr-1) Antibody (#108428 Biolegend) [1:200]
38) Anti-mouse/human n CD11b Antibody (#101259 Biolegend) [1:200]
39) Anti-mouse CD41a Monoclonal Antibody (eBioMWReg30 (MWReg30), FITC (#11-0411-82 ebiosciences) [1:200]
40) Anti-mouse/rat CD61 CD61 Antibody (#104318 Biolegend) [1:200]
41) Anti-mouse/human CD45R/B220 Antibody (#103223 Biolegend) [1:200]
42) Anti-mouse CD90.2 (Thy-1.2) Antibody (#140318 Biolegend) [1:200]

Validation

The antibodies used in this study are widely used and well validated in the literature.
1) https://www.rndsystems.com/products/mouse-il-1-ri-antibody_af771
2) https://www.cellsignal.com/products/primary-antibodies/phospho-p38-mapk-thr180-tyr182-antibody/9211
3) https://www.cellsignal.com/products/primary-antibodies/phospho-nf-kb-p65-ser536-93h1-rabbit-mab/3033?site-search-type=Products&N=4294956287&Ntt=%233033%2C&fromPage=plp&_requestid=1231874
4) https://www.cellsignal.com/products/primary-antibodies/phospho-sapk-jnk-thr183-tyr185-antibody/9251
5) https://www.cellsignal.com/products/primary-antibodies/phospho-smad2-ser465-467-138d4-rabbit-mab/3108?gclid=Cj0KCQjwheyUBhD-ARIsAHJNM-P6P_mI7_PeCJ_jPB7Ti4u3KWojLAVHvh3vFthb6pkRI41O-zFAKtkaAlC9EALw_wcB&gclsrc=aw.ds
6) https://www.cellsignal.com/products/primary-antibodies/p38-mapk-antibody/9212?site-search-type=Products&N=4294956287&Ntt=%239212&fromPage=plp&_requestid=1232046
7) https://datasheets.scbt.com/sc-372.pdf
8) https://www.scbt.com/p/jnk1-3-antibody-c-17
9) https://www.cellsignal.com/products/primary-antibodies/smad2-d43b4-xp-rabbit-mab/5339
10) https://www.sigmaaldrich.com/US/en/substance/monoclonalantibactinantibodyproducedinmouse1234598765?gclid=Cj0KCQjwxdSHBhCdARIsAG6zhlV07km_QAOWkNMTnvAnITGMut6qI4yBGve4jonCkGXArJPTzUJy8ZQaAgmMEALw_wcB
11) https://www.abcam.com/collagen-iii-antibody-ab7778.html
12) https://www.jacksonimmuno.com/catalog/products/111-025-003
13) https://www.thermofisher.com/antibody/product/CD3e-Antibody-clone-145-2C11-Monoclonal/45-0031-82
14) https://www.biolegend.com/en-us/products/percp-cyanine5-5-anti-mouse-cd19-antibody-13640
15) https://www.thermofisher.com/antibody/product/CD8a-Antibody-clone-53-6-7-Monoclonal/45-0081-82
16) https://www.biolegend.com/en-us/products/percp-cyanine5-5-anti-mouse-cd19-antibody-13640
17) https://www.thermofisher.com/antibody/product/CD45R-B220-Antibody-clone-RA3-6B2-Monoclonal/45-0452-82
18) https://www.biolegend.com/en-us/products/percp-cyanine5-5-anti-mouse-ly-6g-ly-6c-gr-1-antibody-4286
19) https://www.biolegend.com/en-us/products/percp-cyanine5-5-anti-mouse-cd127-il-7ralpha-antibody-6196
20) https://www.biolegend.com/en-us/products/percp-cyanine5-5-anti-mouse-ter-119-erythroid-cells-antibody-4292
21) https://www.biolegend.com/en-us/products/apc-cyanine7-anti-mouse-cd117-c-kit-antibody-5905
22) https://www.thermofisher.com/antibody/product/Ly-6A-E-Sca-1-Antibody-clone-D7-Monoclonal/25-5981-82
23) https://www.thermofisher.com/antibody/product/CD135-Flt3-Antibody-clone-A2F10-Monoclonal/17-1351-82
24) https://www.thermofisher.com/antibody/product/CD34-Antibody-clone-RAM34-Monoclonal/11-0341-82
25) https://www.thermofisher.com/antibody/product/CD16-CD32-Antibody-clone-93-Monoclonal/12-0161-82
26) https://www.thermofisher.com/antibody/product/F4-80-Antibody-clone-BM8-Monoclonal/17-4801-82
27) https://www.thermofisher.com/antibody/product/CD16-CD32-Antibody-clone-93-Monoclonal/12-0161-82
28) https://www.biolegend.com/en-us/products/percp-cyanine5-5-anti-mouse-ly-6g-ly-6c-gr-1-antibody-4286

29) https://www.biolegend.com/en-us/products/brilliant-violet-785-anti-mouse-human-cd11b-antibody-7958
30) https://www.biolegend.com/en-us/products/fitc-anti-mouse-ter-119-erythroid-cells-antibody-1865
31) https://www.biolegend.com/en-us/products/pe-cyanine7-anti-mouse-cd71-antibody-6185
32) https://www.thermofisher.com/antibody/product/CD45R-B220-Antibody-clone-RA3-6B2-Monoclonal/56-0452-82
33) https://www.biolegend.com/en-us/products/brilliant-violet-605-anti-mouse-tcr-beta-chain-antibody-13533
34) https://www.biolegend.com/en-us/products/apc-cyanine7-anti-mouse-cd41-antibody-13014
35) https://www.biolegend.com/en-us/products/apc-anti-mouse-ter-119-erythroid-cells-antibody-1863

36) https://www.biolegend.com/en-us/products/pe-anti-mouse-cd71-antibody-1631
37) https://www.biolegend.com/en-us/products/percp-cyanine5-5-anti-mouse-ly-6g-ly-6c-gr-1-antibody-4286
38) https://www.biolegend.com/en-us/products/brilliant-violet-650-anti-mouse-human-cd11b-antibody-7638
39) https://www.thermofisher.com/antibody/product/CD41a-Antibody-clone-eBioMWReg30-MWReg30-Monoclonal/11-0411-82
40) https://www.biolegend.com/en-us/products/pe-cyanine7-anti-mouse-rat-cd61-antibody-14671
41) https://www.biolegend.com/en-us/products/apc-cyanine7-anti-mouse-human-cd45r-b220-antibody-1938
42) https://www.biolegend.com/en-us/products/brilliant-violet-605-anti-mouse-cd90-2-thy-1-2-antibody-7866

Animals and other organisms

Policy information about studies involving animals; ARRIVE guidelines recommended for reporting animal research

Laboratory animals	All mice were bred and maintained under a pathogen-free, 12-hour light/dark cycle environment. All experiments were conducted with age- and sex-matched mice in a C57BL/6 background. 6-8 weeks old mice were used for our experiments.<br>Conditional Jak2V617F knock-in mice were described in our previous studies (Akada et. al., 2010). Mx1Cre (Kuhn et. al. 1995; JAX Cat# 003556), IL-1R1 floxed (Robson et. al., 2016; JAX Cat# 028398), Prx1Cre (Logan et. al., 2002; JAX Cat# 005584) and UBC-GFP (Schaefer et. al., 2001; JAX Cat# 004353) mice were previously described and obtained from the Jackson Laboratory.
Wild animals	No wild animals were used in the study.
Field-collected samples	No field-collected samples were used in this study.
Ethics oversight	Animal experiments were performed under the study protocol 4190 as approved by the University of Virginia Institutional Animal Care and Use Committee.

Note that full information on the approval of the study protocol must also be provided in the manuscript.

Human research participants

Policy information about studies involving human research participants

Population characteristics	Peripheral blood samples from MPN patients were collected at University of Virginia Cancer Center.<br>All participants were adults, more than 18 years old. Both male and female MPN patients were included.
Recruitment	Participants were recruited in an unbiased manner.
Ethics oversight	Informed consent was obtained for sample collection according to the protocols approved by the institutional review board of the University of Virginia Health System and in accordance with the Declaration of Helsinki. University of Virginia ethics committee approved the study and the use of anonymous archived samples.

Note that full information on the approval of the study protocol must also be provided in the manuscript.

Flow Cytometry

Plots

Confirm that:

- [x] The axis labels state the marker and fluorochrome used (e.g. CD4-FITC).
- [x] The axis scales are clearly visible. Include numbers along axes only for bottom left plot of group (a 'group' is an analysis of identical markers).
- [x] All plots are contour plots with outliers or pseudocolor plots.
- [x] A numerical value for number of cells or percentage (with statistics) is provided.

Methodology

Sample preparation	Single-cell suspensions were prepared from BM and spleen, and red blood cells were lysed with red blood cell lysis solution. Cells were washed and resuspended in PBS plus 2% FBS and stained for 30min on ice with directly conjugated monoclonal antibody specific for Ter119, CD71, CD41, CD61, Mac-1, Gr-1, B220, or TCRβ. For HSC/progenitor analysis, BM cells were stained for 60min on ice with antibody against c-Kit, Sca-1, Flk2 (CD135), CD34, CD16/32 (FcγR II/III), and antibody against lineage (Lin) markers including CD3e, CD4, CD8, CD19, B220, Gr-1, Ter119, and IL-7R (CD127). To distinguish between donor-derived and recipient hematopoietic cells in competitive bone marrow transplantation experiments, PE-CD45.1 and FITC-CD45.2 conjugated antibodies were used for flow cytometry.

Instrument	Data were collected on Cytek Aurora or Cytek Northern Lights (Cytek Biosciences).
Software	Data were analyzed using FlowJo software version 10 (FlowJo, LLC).
Cell population abundance	Purity of the isolated samples was obtained by antibody stain and FACS sorting. Sample purity was greater than 90%.
Gating strategy	LSK (Lin– Sca1+ c-kit+), LT-HSC (Lin- Sca1+ c-kit+ CD34- CD135-) ST-HSC (Lin- Sca1+ c-kit+ CD34+ CD135-), LK (Lin-c-kit+), CMP (Lin-c-kit+CD34+CD32/16lo), GMP (Lin-c-kit+CD34+CD32/16hi), MEP (Lin-c-kit+CD34-CD32/16-)

☒ Tick this box to confirm that a figure exemplifying the gating strategy is provided in the Supplementary Information.
